# Genetic Deficiency of MicroRNA‐15a/16‐1 Confers Resistance to Neuropathological Damage and Cognitive Dysfunction in Experimental Vascular Cognitive Impairment and Dementia

**DOI:** 10.1002/advs.202104986

**Published:** 2022-04-11

**Authors:** Chao Zhou, Ping Sun, Yang Xu, Yuang Chen, Yixian Huang, Milton H. Hamblin, Lesley Foley, T. Kevin Hitchens, Song Li, Ke‐Jie Yin

**Affiliations:** ^1^ Pittsburgh Institute of Brain Disorders & Recovery Department of Neurology University of Pittsburgh School of Medicine Pittsburgh PA 15213 USA; ^2^ Center for Pharmacogenetics University of Pittsburgh School of Pharmacy Pittsburgh PA 15213 USA; ^3^ Tulane University Health Sciences Center Tulane University New Orleans LA 70112 USA; ^4^ Animal Imaging Center Department of Neurobiology University of Pittsburgh School of Medicine Pittsburgh PA 15203 USA; ^5^ Geriatric Research Education and Clinical Center Veterans Affairs Pittsburgh Healthcare System Pittsburgh PA 15240 USA

**Keywords:** AKT3, grey matter lesions, IL‐10RA, microRNAs, miR‐15a/16‐1, vascular cognitive impairment and dementia, white matter lesions

## Abstract

Chronic cerebral hypoperfusion‐derived brain damage contributes to the progression of vascular cognitive impairment and dementia (VCID). Cumulative evidence has shown that microRNAs (miRs) are emerging as novel therapeutic targets for CNS disorders. In this study, it is sought to determine the regulatory role of miR‐15a/16‐1 in VCID. It is found that miR‐15a/16‐1 knockout (KO) mice exhibit less cognitive and sensorimotor deficits following VCID. Genetic deficiency of miR‐15a/16‐1 in VCID mice also mitigate myelin degeneration, axonal injury, and neuronal loss. Mechanistically, miR‐15a/16‐1 binds to the 3’‐UTR of AKT3 and IL‐10RA. Genetic deletion of miR‐15a/16‐1 increases AKT3 and IL‐10RA expression in VCID brains, and intranasal delivery of AKT3 and IL‐10RA siRNA‐loaded nanoparticles partially reduce brain protection and cognitive recovery in miR‐15a/16‐1 KO mice after VCID. In conclusion, the miR‐15a/16‐1‐IL/10RA/AKT3 axis plays a critical role in regulating vascular brain damage and cognitive decline after VCID. Targeting miR‐15a/16‐1 is a novel therapeutic approach for the treatment of VCID.

## Graphical abstract

Genetic deletion of miR‐15a/16‐1 promotes the recovery of neurobehavioral functions and alleviates cerebral white and grey matter injury after vascular cognitive impairment and dementia (VCID). Mechanistically, deficiency of the miR‐15a/16‐1 gene preserves AKT3 and IL‐10RA activity in VCID mice. Intranasal delivery of AKT3 and IL‐10RA siRNA‐loaded nanoparticles partially blocks brain protection and functional recovery in miR‐15a/16‐1 KO mice after VCID.

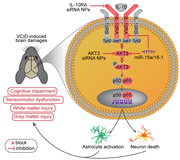

## 1 Introduction

Vascular cognitive impairment and dementia (VCID) is the second common reason for dementia with variable subtypes and inconsistent diagnostic criteria.^[^ ^1^ ^]^ Currently, identifiable or tractable therapeutic targets, as well as promising clinical trials for VCID, are still lacking. Some drugs used for patients with Alzheimer's dementia, such as memantine and cholinesterase inhibitors, have shown to be ineffective in the treatment of VCID.^[^ ^2^ ^]^ These failed clinical trials indicate the complexity of elucidating appropriate therapeutic targets in VCID.

Up to now, the pathogenesis of VCID is still not fully understood. VCID is a cerebrovascular disease with complicated etiological and pathological changes. Studies have shown that numerous neuropathological changes may collectively contribute to the cognitive impairment in vascular dementia, including decreased cerebral blood flow (CBF), insufficient cerebral oxygen supply, chronic inflammation, subcortical infarctions, white matter (WM) damage, and neuronal death.^[^ ^1^, ^3^ ^]^ Once CBF is reduced, the vessels in WM are more vulnerable to suffer ischemic and hypoxic destruction.^[^ ^4^ ^]^ Long‐term chronic cerebral hypoperfusion (CCH) induces neuronal axon fibers in the WM to deform and degenerate and reduce the fractional anisotropy (FA) value in diffusion tensor imaging (DTI).^[^ ^5^ ^]^ Injured WM blocks nerve conduction and leads to cognitive impairment and memory loss.^[^ ^6^ ^]^ Experimental VCID animals showed decreased axonal thickness and small infarctions in the external capsule (EC).^[^ ^5c^ ^]^ Long‐term chronic hypoperfusion can also result in the reduction of tight junction proteins ZO‐1, occludin, and claudin‐5^[^ ^7^ ^]^ that causes BBB dysfunction and leakage, and subsequent neuroinflammatory responses. CCH also triggers the production of pro‐apoptotic factors, pro‐inflammatory factors, and reactive oxygen species (ROS) in neurons, leading to neuronal loss.^[^ ^8^ ^]^ The injured WM/grey matter areas are ultimately infiltrated by proliferating glial cells,^[^ ^4^ ^]^ which not only deteriorate the neuroinflammatory response, but also interrupt the connection between neurons. It is worth noting that these pathological elements do not develop individually but promote each other.^[^ ^9^ ^]^

MicroRNAs (miRs) negatively regulate the downstream target genes via binding to the 3’‐untranslated regions (3'‐UTR) of their mRNAs. MiRs are involved in a wide range of biological activities, including cell proliferation, differentiation, metabolism, apoptosis, immune responses, and other pathophysiological processes. Studies have shown that miR‐409‐5p, miR‐132, and miR‐10b‐3p were downregulated, while miR‐451a, miR‐222, miR‐486‐5p, and miR‐502‐3p were increased in various biofluids of VCID.^[^ ^4^, ^10^ ^]^ These pathologically‐altered miRs could serve as potential biomarker candidates for the pathogenesis of VCID.^[^ ^11^ ^]^ Moreover, Chen et al. demonstrated that knockdown of miR‐195 prevented the neuropathology and neurodegeneration in VCID.^[^ ^12^ ^]^ Other studies reported that downregulation or genetic deletion of miR‐96, miR‐9, miR‐132, and miR‐210‐5p ameliorated VCID‐related neurobehavior and pathologies.^[^ ^13^ ^]^ In contrast, downregulation or genetic deficiency of miR‐26b, miR‐126, miR‐195, and miR‐181c aggravated VCID outcomes.^[^ ^14^ ^]^

Our previous studies have demonstrated that systematic inhibition or genetic deletion of miR‐15a/16‐1 ameliorates sensorimotor dysfunction and neuroinflammation after ischemic stroke.^[^ ^15^ ^]^ Endothelium‐targeted deletion of miR‐15a/16‐1 not only promoted post‐stroke angiogenesis by enhancing the expression of vascular endothelial growth factor A (VEGFA), fibroblast growth factor 2 (FGF2), vascular endothelial growth factor receptor 2 (VEGFR2), and fibroblast growth factor receptor 1 (FGFR1), but also ameliorates post‐ischemic blood‐brain barrier dysfunction via up‐regulating claudin‐5 expression. Moreover, mice with endothelial deficiency of miR‐15a/16‐1 also show improved long‐term sensorimotor and cognitive functions after ischemic stroke.^[^ ^16^ ^]^ These studies indicated the potential of miR‐15a/16‐1 as a therapeutic target for ischemic stroke and other cerebrovascular diseases. However, whether suppression of miR‐15a/16‐1 can affect the pathological progression of vascular dementia remains unexplored.

In this study, we reveal a proof‐of‐concept for the miR‐15a/16‐1/IL‐10RA/AKT3 axis in VCID. We anticipate that these results will provide evidence for the future therapeutic potentiality of VCID by targeting miR‐15a/16‐1.

## 2 Results

### 2.1 Genetic Deletion of miR‐15a/16‐1 Mitigates CBF Decline in Mice after VCID

**Figure 1:**
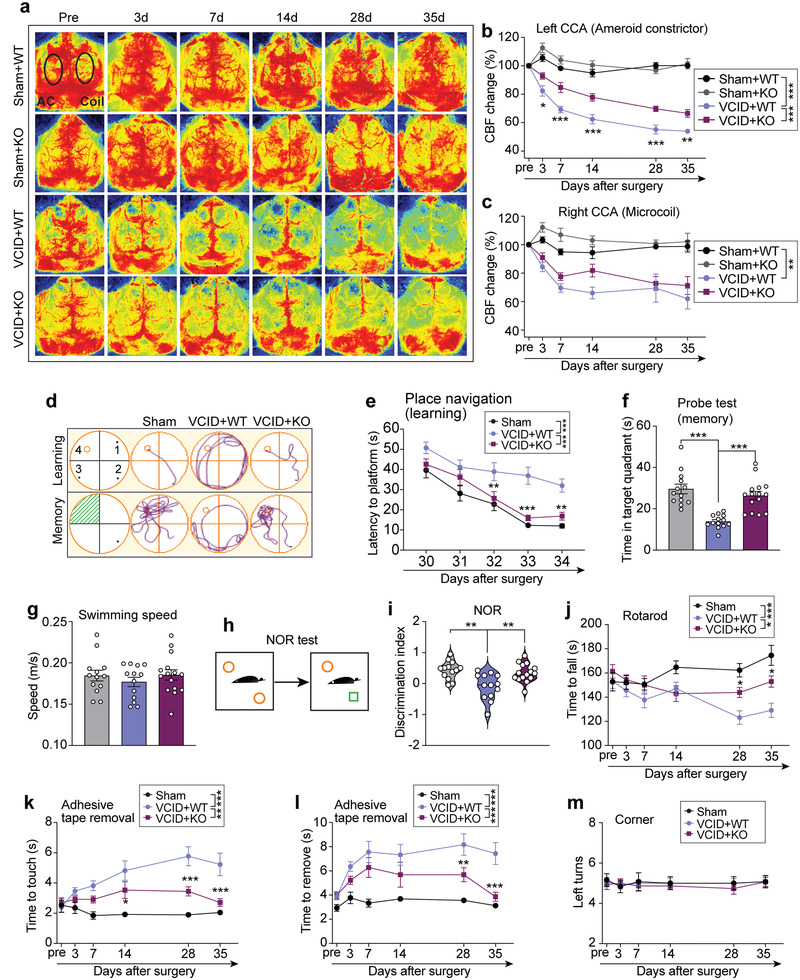
Genetic deletion of miR‐15a/16‐1 promotes long‐term recovery of regional CBF, sensorimotor, and cognitive function in an experimental mouse VCID model. Experimental VCID was induced in miR‐15a/16‐1 KO and WT mice, followed by a 35 d survival period. a) Cortical CBF was monitored in mice using laser speckle imaging before surgery (pre), 3 d, 7 d, 14 d, 28 d, and 35 d after surgery. Representative CBF images (black ellipses: ROIs) were presented. b,c) Quantitative analysis of regional CBF changes in the left and right hemispheres in sham and VCID mice (n = 5–6/group). VCID mice and sham controls were subjected to MWM and NOR tests for examination of long‐term cognitive decline or deficits. d) Swim paths of learning and memory phases during the MWM test. e) The latency to find the hidden platform in the place navigation phase (learning). f) The swim time in the target quadrant in the probe test (memory). g) Average swimming speed in the MWM test (n = 12–15/group). h) Graphic protocol of NOR test. i) The discrimination index at test phase of NOR (n = 12–15/group). Sensorimotor function was examined in VCID mice and sham controls at the indicated time points (−1, 3, 7, 14, 28, and 35 d after operation, n = 13–15/group). j) The time to fall in the rotarod test. k) The time to touch and l) time to remove the tape in the adhesive tape removal test. m) The left turn numbers in the corner test. Data are presented as mean ± SEM. ^*^ *p* < 0.05, ^**^ *p* < 0.01, and ^***^ *p* < 0.001 versus VCID+WT group. Statistical analyses were performed by one‐way ANOVA and Bonferroni's test (g, h, j) and two‐way ANOVA with Bonferroni's test (b, c, e, j–m).

The overall graphic schedule for in vivo experimental design is shown in Figure S1, Supporting Information. Asymmetric common carotid artery surgery (ACAS) was conducted to induce experimental VCID in mice by implantation of an ameroid constrictor (AC) on the left common carotid artery (CCA) and a microcoil on the right CCA.^[^ ^6b^ ^]^ MiR‐15a/16‐1 KO, and wild‐type (WT) mice were subjected to either a VCID or sham operation and CBF changes were monitored continuously before and 3, 7, 14, 28, and 35 d after VCID (**Figure** **1**a) by laser speckle imaging. No significant differences were detected between WT and miR‐15a/16‐1 KO mice under sham conditions. The relative CBF value gradually declined from 3 d to 35 d of VCID in the hemisphere at both the AC side and microcoil side (Figure 1b, c). Genetic deletion of miR‐15a/16‐1 significantly mitigated the CBF decline in the AC side of the hemisphere, as evidenced by a higher relative CBF value than WT mice at 3 d, 7 d, 14 d, 28 d, and 35 d after VCID. No significant difference of CBF change was observed in the microcoil side of the hemisphere between miR‐15a/16‐1 KO and WT mice after VCID.

To investigate the mechanism of CBF improvement in miR‐15a/16‐1 KO mice after VCID, miR‐15a/16‐1 KO, and WT mice were subjected to either a VCID or sham operation. After 14 d of operation, angiogenesis‐related target genes of miR‐15a/16‐1, VEGFA and FGF2, were examined by western blotting (Figures S2 and S3, Supporting Information). The quantitative data (Figure S2b, c, Supporting Information) showed that the protein expression levels of VEGFA and FGF2 were significantly decreased in WT mice after 14 d of VCID. In contrast, genetic deletion of miR‐15a/16‐1 dramatically increased FGF2 and VEGFA expression in VCID brains in comparison with WT controls. These data suggest that genetic deficiency of miR‐15a/16‐1 may enhance cerebral VEGFA and FGF2 expression to trigger pro‐angiogenesis activity and thus improve CBF in mice after VCID.

### 2.2 Genetic Deletion of miR‐15a/16‐1 Alleviates Long‐Term Sensorimotor and Cognitive Dysfunction in Mice after VCID

We further investigated whether miR‐15a/16‐1 genetic deletion affects neurobehavioral (cognitive and sensorimotor) functions in VCID mice. The Morris water maze (MWM) test and novel objective recognition (NOR) test were conducted to assess cognitive function. There was no difference in neurobehavioral function between sham‐operated miR‐15a/16‐1 KO and WT mice (Figure S4a–g, Supporting Information). Thus, the sham groups of miR‐15a/16‐1 KO, and WT mice were combined together for neurobehavioral evaluation. Sham‐operated mice had normal spatial learning ability as evidenced by a continuously decreased time to locate the platform, whereas VCID‐induced experimental mice (VCID+WT group) required a longer time to find the platform in the learning phase (Figure 1d, e). Interestingly, the VCID‐induced miR‐15a/16‐1 KO group showed a shorter time to find the platform compared with WT mice after VCID, suggesting an improved long‐term spatial learning ability. Reference memory was also diminished in WT mice after VCID, as shown by less time spent in the target quadrant during the probe test (Figure 1f). Genetic deletion of miR‐15a/16‐1 significantly improved reference memory after VCID, as miR‐15a/16‐1 KO mice showed longer time spent in the target quadrant in comparison with WT mice (Figure 1F). There were no significant differences in swimming speed among all experimental groups (Figure 1g), suggesting that learning and memory outcomes were not affected by swimming speed. The NOR test (Figure 1h) demonstrated that sham‐operated mice exhibited a higher discrimination index (Figure 1i). After VCID, WT mice presented a decreased discrimination index, suggesting impaired memory and recognition capability. But mice with genetic deletion of miR‐15a/16‐1 showed improved recognition capability following VCID, showing a higher discrimination index (Figure 1i).

To evaluate sensorimotor functions, rotarod test, adhesive tape removal test, and corner test were conducted in miR‐15a/16‐1 KO and WT mice before and after 3, 7, 14, 28, and 35 d of operation. As demonstrated in Figure 1j–m, sensorimotor dysfunction progressed slowly and gradually followed the onset of VCID. We found that miR‐15a/16‐1 KO mice presented better sensorimotor function compared with WT mice after VCID as evidenced by increased staying time on the rotarod (Figure 1j) and a shorter time to touch and remove the adhesive tapes in the adhesive tape removal test (Figure 1k, l). No unilateral abnormalities of sensorimotor function were detected in VCID mice in the corner test, as indicated by the tantamount turns (Figure 1m). No significant differences in body weight and survival rate were observed between miR‐15a/16‐1 KO mice and WT controls after VCID (Figure S5a–c, Supporting Information). Of note, the body weights of miR‐15a/16‐1 KO and WT mice gradually decreased following VCID and declined until 14 d after VCID, and then gradually increased in these experimental mice after 14 d VCID (Figure S5b, Supporting Information). The excluded experimental animals in this study are listed in Figure S5d, Supporting Information.

Taken together, these data demonstrated that genetic deletion of miR‐15a/16‐1 alleviated sensorimotor deficits and cognitive impairments after VCID.

### 2.3 Genetic Deletion of miR‐15a/16‐1 Preserves Myelin Integrity and Reduces Demyelination in Mice after VCID

**Figure 2:**
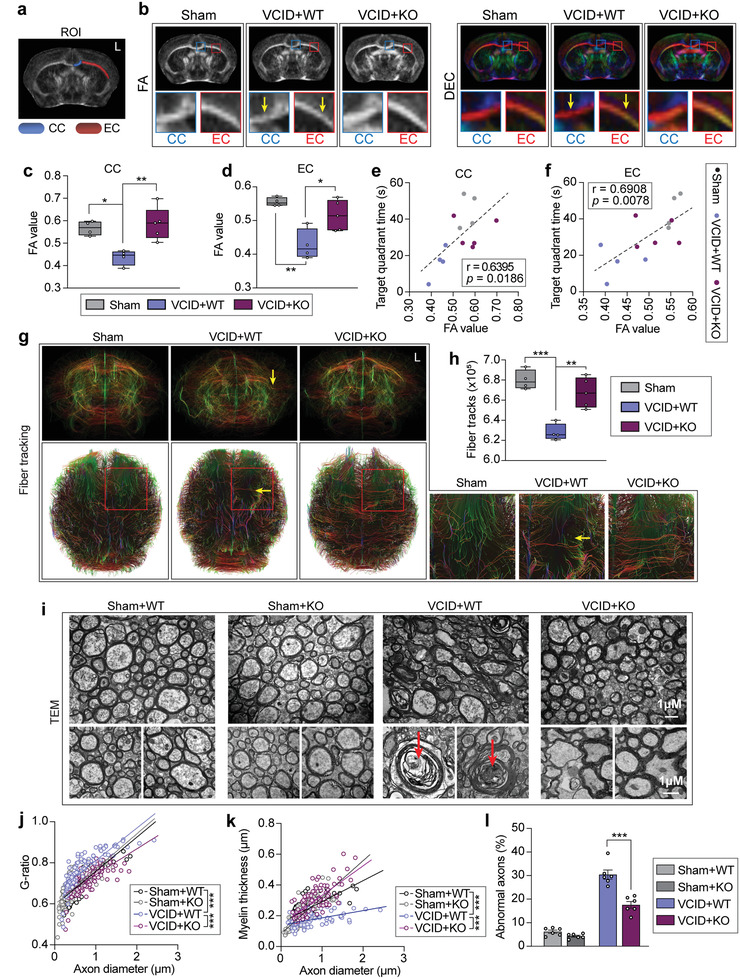
Genetic deletion of miR‐15a/16‐1 preserves long‐term WM integrity in mice after VCID. DTI and TEM were conducted to evaluate the integrity of WM in miR‐15a/16‐1 KO and WT mice 35 d after VCID. a) Image showing the ROI for DTI analysis. b) Representative DTI axial views of FA maps and DEC maps of the same brains (blue and red squares: enlarged images of the CC and EC, respectively; yellow arrows: injured area). c,d) Quantitative analysis of FA values in the CC and EC (n = 4–5/group, one‐way ANOVA & Bonferroni's test). e,f) The correlation analysis of target quadrant time in the MWM test and FA values in the CC and EC areas (n = 4–5/group, Pearson correlation analysis). g) Representative fiber tracking images in mouse brain and h) quantitative analysis of fiber intensity. The brain region of fiber loss was indicated by yellow arrows. i) Representative TEM images showing the ultrastructure of axons and myelin sheaths (red arrows: myelin debris). Quantitative analysis of j) g‐ratio, k) myelin thickness (n = 100 axons/group, simple linear regression and slopes comparison), and l) abnormal axons (one‐way ANOVA & Bonferroni's test) in the CC/EC areas. Data are presented as mean ± SEM or scatterplots. ^*^ *p*<0.05, ^**^ *p*<0.01, or ^***^ *p*<0.001 versus VCID+WT group.

Cerebral WM plays a critical role in maintaining sensorimotor and cognitive functions.^[^ ^4^ ^]^ In this study, DTI was performed to evaluate WM integrity in mice 35 d after VCID. Since no significant difference was detected in sham‐operated WT and miR‐15a/16‐1 KO mice via DTI scans, these two groups were combined as one sham group for DTI statistical analysis. Region of interests (ROIs) in DTI analyses are shown in **Figure** **2**a. DTI and quantitative data showed that WT mice exhibited significantly decreased FA values (Figure 2b) in the corpus callosum (CC) and EC (Figure 2c, d) 35 d after VCID, indicating disrupted myelin integrity. Genetic deletion of miR‐15a/16‐1 significantly ameliorated WM injury after VCID, showing a higher FA value in the CC and EC versus WT controls (Figure 2c, d). Next, correlation analysis between FA values and target quadrant times (MWM test) was conducted in sham‐operated and VCID mice with or without genetic deletion of miR‐15a/16‐1 to determine whether WM injury was related to cognitive impairment. As shown in Figure 2e,f, FA values in the CC and EC were positively correlated with target quadrant times, suggesting that intact WM is vital to cognitive function in VCID mice. The fibers in mouse brains were tracked and reconstructed by using DSI Studio software (Figure 2g). The fiber intensity was reduced in mouse brains after VCID. However genetic deletion of miR‐15a/16‐1 significantly increased the fiber numbers after VCID (Figure 2h).

To evaluate the ultrastructure of the myelin sheath and axons, the CC/EC tissues were collected from miR‐15a/16‐1 KO and WT mice 35 d after VCID or sham operation and examined by transmission electron microscopy (TEM). As shown in Figure 2i, there were no significant ultrastructural changes in myelin and axons between miR‐15a/16‐1 KO and WT mice under sham‐operated conditions. In comparison with sham controls, VCID mice showed evident myelin loss and abnormal axons defined as axolysis and/or dense axoplasms in the CC/EC. Also, ACAS caused the accumulation of myelin debris in the CC/EC (Figure 2i). However, miR‐15a/16‐1 genetic deletion in mice significantly improved the ultrastructure of myelin in comparison with WT controls 35 d after VCID (Figure 2i), as further evidenced by quantitative analysis revealing declined G‐ratios of individual fibers in relation to respective axon diameters (Figure 2j), increased myelin thickness (Figure 2k), and reduced numbers of abnormal axons (Figure 2l).

**Figure 3:**
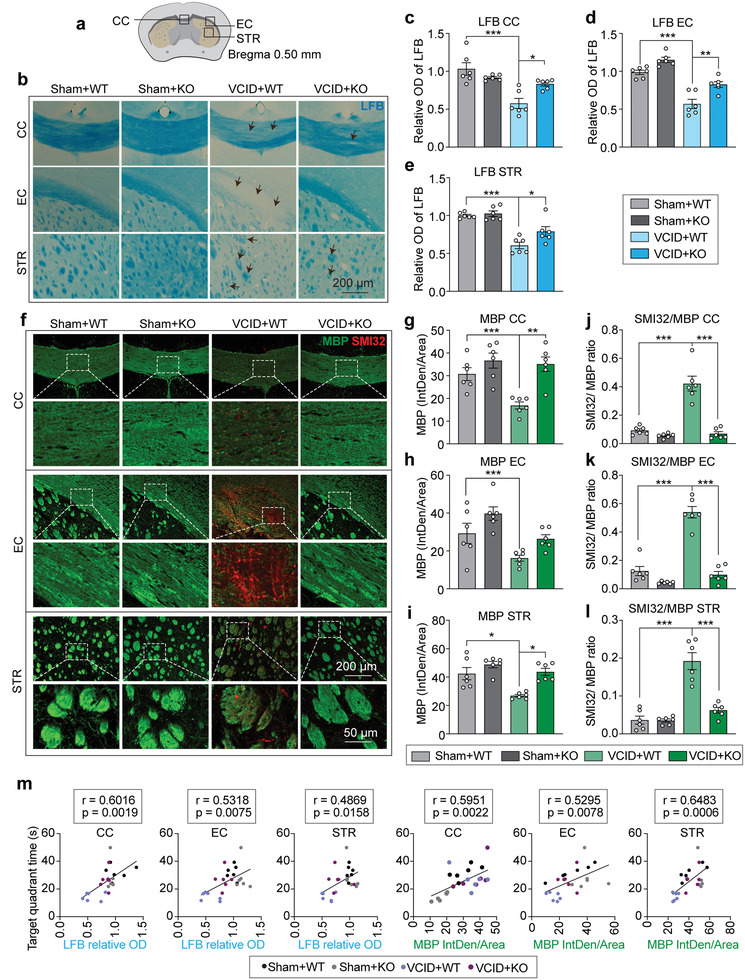
MiR‐15a/16‐1 genetic deletion protects against long‐term myelin loss and axonal damage in mice after VCID. LFB histological staining and MBP/SMI32 immunofluorescence double staining were applied to miR‐15a/16‐1 KO and WT mouse brains harvested 35 d after VCID to detect myelin loss and axonal damage. a) Coordinates of LFB and MBP/SMI32 staining and brain regions for analysis. b) Representative LFB images of the CC, EC, and STR areas (Black arrow: demyelinated area). c–e) Quantitative analysis of relative OD values of LFB in the CC, EC, and STR areas (n = 6/group, one‐way ANOVA & Bonferroni's test) f) Representative images of MBP (green) and SMI32 (red) double‐immunostaining in the CC, EC, and STR areas. g–i) Quantitative analysis of MBP fluorescence intensities and j–l) the ratio of SMI32/MBP in the CC, EC, and STR areas (n = 6/group, one‐way ANOVA & Bonferroni's test). m) Correlation analysis of target quadrant time in the MWM test and LFB or MBP staining of the CC, EC, and STR areas (n = 6/group, Pearson correlation analysis). Data are presented as mean ± SEM. ^*^ *p*<0.05, ^**^ *p*<0.01, or ^***^ *p*<0.001 versus VCID+WT group.

We further confirmed the above DTI and TEM results by Luxol fast blue (LFB) histological staining and myelin basic protein (MBP, myelin marker)/neurofilament heavy polypeptide (clone: SMI32, injured axon marker) double‐immunofluorescence (IF) staining in the CC, EC, and striatum after 35 d of VCID. The imaging regions of WM staining are shown in **Figure** **3**a. We observed a significant loss of myelin in the CC, EC, and striatum in WT mice 35 d after VCID. Genetic deletion of miR‐15a/16‐1 significantly ameliorated the demyelination in mice after VCID (Figure 3b) and presented a higher relative OD value of LFB in the CC, EC, and striatum (Figure 3c–e). Immunostaining results demonstrated myelin loss accompanied by axonal damages in the WT group after 35 d of VCID (Figure 3f, Figure S6a, Supporting Information), showing significantly decreased MBP and increased SMI32 expression (Figure 3f–i, Figure S6a–d, Supporting Information). Interestingly, genetic deletion of miR‐15a/16‐1 significantly preserved MBP and reduced SMI32 in the CC, EC, and striatum (Figure 3f–i, Figure S36d, Supporting Information). Subsequent quantitative analyses showed that the ratio of SMI32/MBP was significantly elevated after VCID and decreased by genetic deletion of miR‐15a/16‐1 (Figure 3j–l). Moreover, correlation analysis between target quadrant time (MWM test) and LFB or MBP staining was performed to determine whether WM integrity was related to cognitive impairment. As shown in Figure 3m, the relative OD of LFB and fluorescence intensities of MBP in the CC, EC, and striatum were positively correlated with target quadrant times, suggesting that WM integrity is essential to cognitive function in VCID mice.

These results indicate that genetic deletion of miR‐15a/16‐1 alleviates the demyelination process and preserves intact WM fibers after VCID.

### 2.4 Genetic Deletion of miR‐15a/16‐1 Ameliorates Long‐Term Grey Matter Injury in Mice after VCID

**Figure 4:**
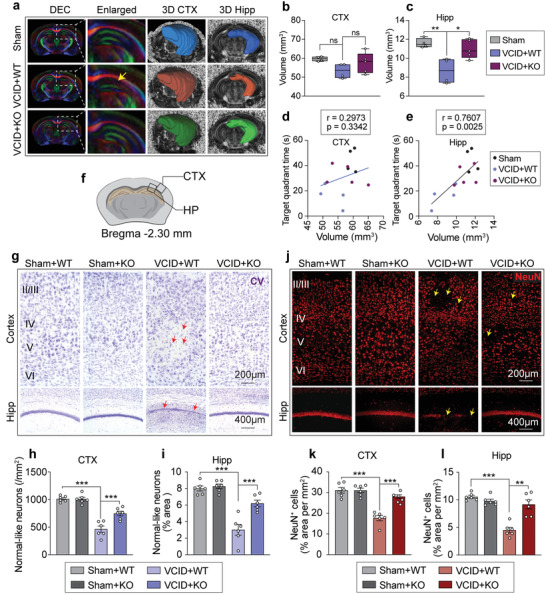
Genetic deletion of miR‐15a/16‐1 reduces neuronal loss and hippocampal atrophy in mice after VCID. a) Hippocampal volume was evaluated with DTI. Representative DEC maps and hippocampal 3D reconstitution images of miR‐15a/16‐1 KO and WT mouse brains 35 d after VCID (white lines: enlarged areas; yellow arrow: atrophy area). b,c) Quantitative analysis of volume in cerebral cortex (CTX) and hippocampus (Hipp) areas (n = 4–5/group, one‐way ANOVA & Bonferroni's test). d,e) The correlation analysis of target quadrant time in the MWM test and volume of the CTX and Hipp (n = 4–5/group, Pearson correlation analysis). In order to evaluate neuronal loss in VCID brains, CV histological staining and NeuN immunofluorescence staining were conducted in miR‐15a/16‐1 KO and WT mouse brains harvested 35 d after VCID. f) Coordinates of CV and NeuN staining and brain regions for analysis. g) Representative images of CV staining in the CTX and Hipp regions (red arrows: pyknotic neuron cells). h,i) Quantitative analysis of normal‐like neurons in the CTX and Hipp areas (n = 6/group, one‐way ANOVA & Bonferroni's test). j) Representative images of NeuN staining in the CTX and Hipp areas (yellow arrows: NeuN‐negative staining areas). k,l) Quantitative analysis of NeuN‐positive neurons in the CTX and Hipp (n = 6/group, one‐way ANOVA & Bonferroni's test). Data are presented as mean ± SEM. ^*^ *p*<0.05, ^**^ *p*<0.01, or ^***^ *p*<0.001 versus VCID+WT group.

VCID is characterized with neuronal loss or atrophy in the cerebral cortex and hippocampus. To assess the volumetric changes of miR‐15a/16‐1 KO and WT mice 35 d after VCID, 3D volumes were segmented from the cerebral cortex and hippocampus (**Figure** **4**a) using DSI Studio software. Compared with sham controls, the volume of the hippocampus (not the cerebral cortex) was significantly reduced in mice 35 d after VCID (Figure 4a–c). Of note, genetic deletion of miR‐15a/16‐1 significantly protected the hippocampus from VCID‐induced volume loss (Figure 4c). Moreover, the volume of hippocampus, but not cerebral cortex, was positively correlated with target quadrant times in the MWM test (Figure 4d, e). Taken together, these data indicate that genetic deletion of miR‐15a/16‐1 protects against neuronal death and hippocampal atrophy in mice after VCID. In addition, no significant difference was detected in brain morphology, brain weight, cerebral microvessel morphology, cortical volume, and hippocampal volume between miR‐15a/16‐1 KO and WT mice under normal conditions (Figure S7a–j, Supporting Information).

We also examined the effect of miR‐15a/16‐1 genetic deletion on neuronal loss in the cerebral cortex and hippocampus by using Cresyl Violet (CV) histological staining and NeuN (neuronal nuclear marker) immunostaining in mice 35 d after VCID. Imaging regions of grey matter staining are shown in Figure 4f. Compared with sham controls, there were many pyknotic dots in the cerebral cortex and hippocampus that were considered dead neurons (Figure 4g) in WT mice 35 d after VCID. Genetic deletion of miR‐15a/16‐1 dramatically reduced neuronal death after VCID, showing almost normal‐like neurons in the cerebral cortex and hippocampus (Figure 4g–i). NeuN immunostaining also confirmed these results. As shown in Figure 4j–l, VCID at 35 d induced a remarkable reduction of NeuN‐positive neurons in the cerebral cortex and hippocampus in WT mice, whereas genetic deletion of miR‐15a/16‐1 significantly increased the survival of NeuN‐positive neurons.

We further examined whether genetic deletion of miR‐15a/16‐1 affects synaptic plasticity in mouse brains after VCID by detecting the levels of synaptic plasticity‐related proteins, PSD‐95, and synaptophysin in cerebral cortex. As shown in Figure S8 & Figure S9, Supporting Information, the protein expression levels of PSD95 and synaptophysin were significantly reduced in WT mice after 14 d of VCID when compared to sham controls. However, genetic deletion of miR‐15a/16‐1 dramatically increased PSD95 and synaptophysin expression in the cerebral cortex after VCID in comparison with WT controls.

### 2.5 Genetic Deletion of miR‐15a/16‐1 Alleviates Astrocyte Activation in Mouse Brains after VCID

**Figure 5:**
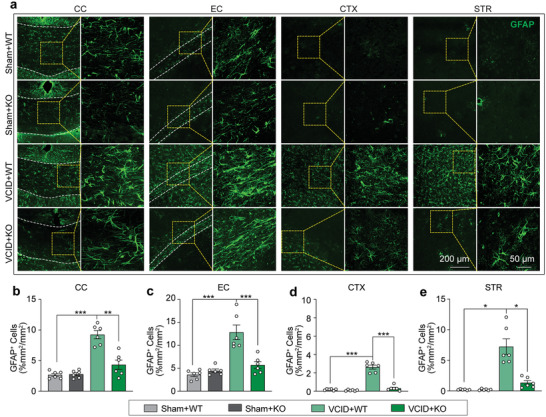
Genetic deletion of miR‐15a/16‐1 alleviates astroglial activation in WM and grey matter in mouse VCID brains. GFAP (astrocyte marker) immunofluorescence staining was conducted in miR‐15a/16‐1 KO and WT mouse brains harvested 3 d after VCID to evaluate the activation of astrocytes in the brain. a) Representative images of GFAP (green) staining in the CC, EC, CTX, and STR areas. b–e) Quantitative analysis of GFAP‐positive cells in the CC, EC, CTX, and STR areas (n = 6/group, one‐way ANOVA & Bonferroni's test). Data are represented as mean ± SEM. ^*^ *p*<0.05, ^**^ *p*<0.01, or ^***^ *p*<0.001 versus VCID+WT group.

As astrocyte‐derived persistent chronic neuroinflammation actively participates in the progression of VCID,^[^ ^4^ ^]^ we also examined astrocyte activation in mouse brains 3 d after VCID. As shown in **Figure** **5**a–e, under sham conditions, there were a moderate number of GFAP‐positive astrocytes in the CC and EC area, but less located in the cerebral cortex and striatum in both miR‐15a/16‐1 KO and WT mice. After 3 d of VCID, significantly higher numbers of astrocytes were present not only in the CC and EC areas but also extended to the cerebral cortex and striatum areas of WT mouse brains, suggesting robust astrocytic activation after 3 d of VCID. However, genetic deletion of miR‐15a/16‐1 significantly suppressed the activation of astrocytes in these brain regions in mice 3 d after VCID (Figure 5a–e). Of note, the morphology of astrocytes was analyzed by Imaris software, and no significant differences were detected between miR‐15a/16‐1 KO and WT mice under normal conditions (Figure S10, Supporting Information).

### 2.6 MiR‐15a/16‐1 Translationally Represses AKT3 and IL‐10RA

To further investigate the underlying mechanism of miR‐15a/16‐1‐mediated vascular brain damage and cognitive decline after VCID, we performed bioinformatics analysis and profiled miR‐15a/16‐1 target genes in the miRDB database (http://www.mirdb.org/). We identified the two highest‐scoring target genes of miR‐15a/16‐1, AKT Serine/Threonine Kinase 3 (AKT3) and interleukin 10 receptor alpha (IL‐10RA), which play important roles in sustaining normal function of WM and grey matter in the brain.^[^ ^17^ ^]^

**Figure 6:**
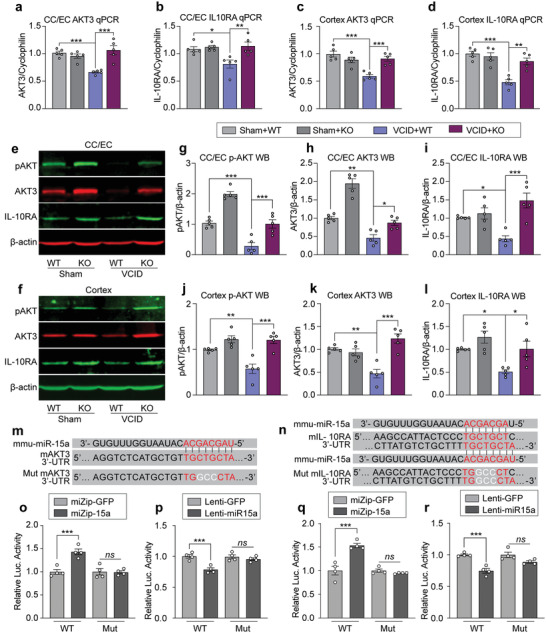
MiR‐15a/16‐1 translationally suppresses AKT3 and IL‐10RA expression in mouse VCID brains. The expression of anti‐inflammatory mediators, AKT3 and IL‐10RA in both WM and grey matter were evaluated in miR‐15a/16‐1 KO and WT mouse brains harvested 3 d after VCID by qPCR and western blotting. a–d) Quantitative analysis of qPCR results showing AKT3 and IL‐10RA relative mRNA expression in the CC/EC and CTX areas (n = 5/group, one‐way ANOVA & Bonferroni's test). e,f) Representative western blotting images of pAKT (Ser473), AKT3, and IL‐10RA in the CC/EC and CTX areas. g–l) Quantitative analysis showing pAKT, AKT3, and IL‐10RA relative protein expression in the CC/EC and CTX areas (n = 5/group, one‐way ANOVA & Bonferroni's test). Data are presented as mean ± SEM. ^*^ *p*<0.05, ^**^ *p*<0.01, or ^***^ *p*<0.001 versus VCID+WT group. m,n) The partial sequence of mature mouse miR‐15a (mmu‐miR‐15a), and its wild‐type and mutated binding sequences on the 3’‐UTR (untranslated regions) regions of mouse AKT3 and IL‐10RA mRNAs. o,p) Quantitative data showing luciferase activity of the reporter vector containing a wild‐type or mutated 3’‐UTR of mouse AKT3 in HEK 293 cells with lentiviral gain‐ or loss‐of‐miR‐15a function by Lenti‐miR‐15a or miZip‐15a. q,r) Quantitative data showing luciferase activity of the reporter vector containing a wild‐type or mutated 3’‐UTR of mouse IL‐10RA in HEK 293 cells with lentiviral gain‐ or loss‐of‐miR‐15a function by Lenti‐miR‐15a or miZip‐15a (n = 4/group, one‐way ANOVA & Bonferroni's test). Data are presented as mean ± SEM. ^***^ *p*<0.001 versus WT+miZip‐GFP or WT+Lenti‐GFP group.

As shown in **Figure** **6**a–d, the mRNA expression levels of AKT3 and IL‐10RA in the CC/EC and cerebral cortex regions were significantly increased in miR‐15a/16‐1 KO mice (Figure 6a–d) 3 d after VCID compared with WT controls. Higher protein levels of AKT3 and IL‐10RA in similar brain regions were also detected in miR‐15a/16‐1 KO mice compared with WT controls 3 d after VCID (Figure 6e–l, Figure S4, Supporting Information). In addition, phospho‐AKT (Ser473) protein expression in the CC/EC and cerebral cortex regions were significantly elevated in miR‐15a/16‐1 KO mice in comparison with WT controls 3 d after VCID (Figure 6e–l, Figure S11, Supporting Information). These results imply that downregulation of AKT3 and IL‐10RA may contribute to VCID‐induced vascular brain damage and cognitive decline, and miR‐15a/16‐1 genetic deletion effectively preserves the expression and function of AKT3 and IL‐10RA in mouse brains.

We further performed dual‐luciferase reporter assays to examine the direct translational regulatory role of miR‐15a/16‐1 in AKT3 and IL‐10RA. As shown in Figure 6m,n, one conserved miR‐15a binding site within the 3’‐UTR of mouse AKT3 mRNA and two conserved miR‐15a binding sites within the 3’‐UTR of IL‐10RA mRNA were found by using bioinformatics analysis, suggesting that miR‐15a may translationally inhibit AKT3 and IL‐10RA through direct interaction with the predicted binding sites. We employed Firefly/Renilla dual‐luciferase reporter plasmids containing a cytomegalovirus‐driven luciferase cDNA fused to wild‐type 3’‐UTRs of AKT3 or IL‐10RA or to 3’‐UTRs with mutated binding sites of the miR‐15a (Figure 6m, n, Figure S12, Supporting Information). HEK 293 cells were infected with miRZip‐15a or Lenti‐miR‐15a to knockdown or overexpress the miR‐15a/16‐1 level for 72 h and then transfected with wild‐type or mutated AKT3 and IL‐10RA luciferase reporter plasmids, respectively. Lentiviral knockdown of miR‐15a significantly increased the luciferase activity of AKT3 and IL‐10RA using wild‐type dual‐luciferase reporter plasmids (Figure 6o, q). In contrast, lentiviral overexpression of miR‐15a/16‐1 significantly decreased the luciferase activity of AKT and IL‐10RA using wild‐type dual‐luciferase reporter plasmids (Figure 6p, r). Neither knockdown nor overexpression of miR‐15a/16‐1 showed any significant changes in luciferase activity when the miR‐15a/16‐1 binding sites at the 3’‐UTRs of AKT3 and IL‐10RA mRNAs were mutated (Figure 6o–r). Collectively, these results indicated that miR‐15a/16‐1 directly binds to the 3’‐UTR regions of mouse AKT3 and IL‐10RA mRNAs and translationally represses their activities.

### 2.7 AKT3 and IL‐10RA are Mainly Located in Neurons and Astrocytes, and their Expression Increased after Genetic Deletion of miR‐15a/16‐1

**Figure 7:**
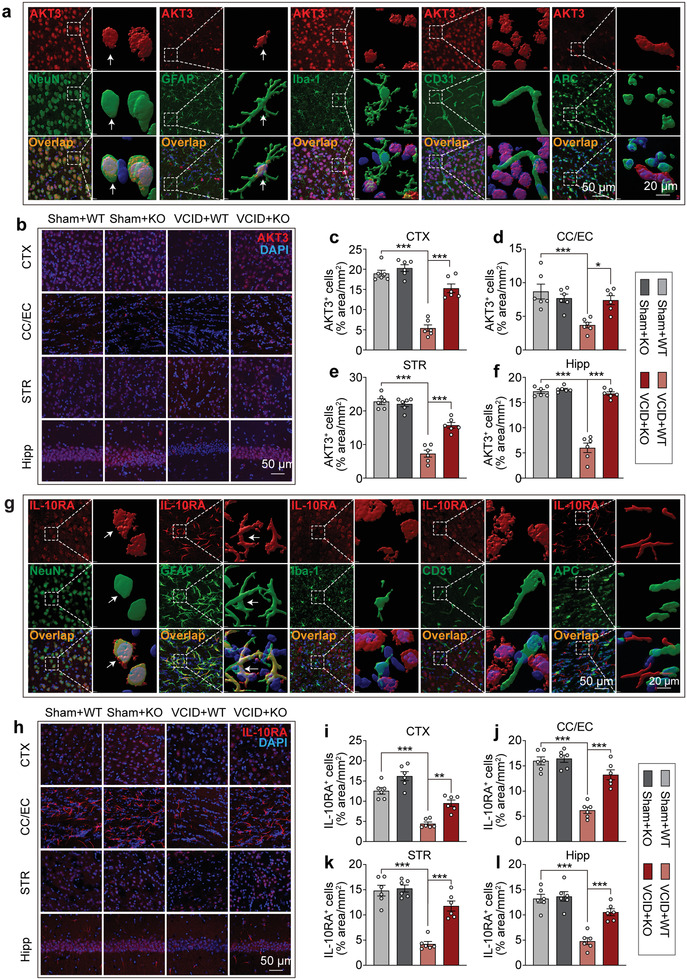
AKT3 and IL‐10RA are mainly expressed in neurons and astrocytes and genetic deletion of miR‐15a/16‐1 rescues AKT3 and IL‐10RA decline in VCID brains. a,g) The expression profiles of AKT3 in different brain cells were detected by double immunostaining of AKT3 (red) with NeuN (neuron marker, green), GFAP (astrocyte marker, green), Iba‐1 (microglia marker, green), CD31 (blood vessel marker, green) and APC (oligodendrocyte marker, green) in sham‐operated mouse brains. 3D reconstruction images showing co‐immunostaining of AKT3/IL‐10RA with NeuN and GFAP (White arrow: co‐immunostained cells). The expression levels of AKT3 and IL‐10RA in different brain regions were also detected in miR‐15a/16‐1 KO and WT mice 3 d after VCID. b,h) Representative images showing immunostaining of AKT3 or IL‐10RA (red) and DAPI (blue) in CTX, CC/EC, STR, and Hipp areas. Quantitative analysis (n = 6/group, one‐way ANOVA & Bonferroni's test) of c–f) AKT3 or i–l) IL‐10RA. Mean fluorescence intensity in both WM (CC/EC, STR) and grey matter (CTX, Hipp). Data are presented as mean ± SEM. ^*^ *p*<0.05, ^**^ *p*<0.01, or ^***^ *p*<0.001 versus VCID+WT group.

Next, we examined the in situ expression of AKT3 and IL‐10RA in different types of brain cells by co‐immunostaining AKT3 or IL‐10RA with several specific neurovascular cell markers, including NeuN (neuron marker), GFAP (astrocyte marker), Iba‐1 (microglia marker), CD31 (endothelial cell marker) and APC (oligodendrocyte cell marker). By using IMARIS 3D reconstruction software, regional 3D reconstruction demonstrated co‐localization of AKT3 or IL‐10RA with NeuN and GFAP (**Figure** **7**a, g), indicating abundant expression of AKT3 and IL‐10RA in neurons and astrocytes. No obvious AKT3 or IL‐10RA expression was observed in microglial cells, endothelial cells, and oligodendrocytes (Figure 7a, g). These data show that AKT3 and IL‐10RA are mainly expressed in neurons and astrocytes.

We also examined the spatial distribution of AKT3 and IL‐10RA in different brain regions. As shown in Figure 7b,h, AKT3 and IL‐10RA are widely distributed in different brain regions, including the cerebral cortex, CC/EC, striatum, and hippocampus. After 3 d of VCID, the expression levels of AKT3 and IL‐10RA were significantly decreased in these brain areas (Figure 7c–f,i–l). However, genetic deletion of miR‐15a/16‐1 increased AKT3 and IL‐10RA expression in these areas when compared with WT controls (Figure 7c–f,i–l). Taken together, these data indicate that miR‐15a/16‐1 genetic deletion rescued the decline of AKT3 and IL‐10RA in both WM and grey matter in mice 3 d after VCID.

### 2.8 Intranasal Delivery of AKT3 and IL‐10RA siRNA‐Loaded Nanoparticles Partially Abolishes Long‐Term Sensorimotor and Cognitive Improvements in miR‐15a/16‐1 KO Mice after VCID

**Figure 8:**
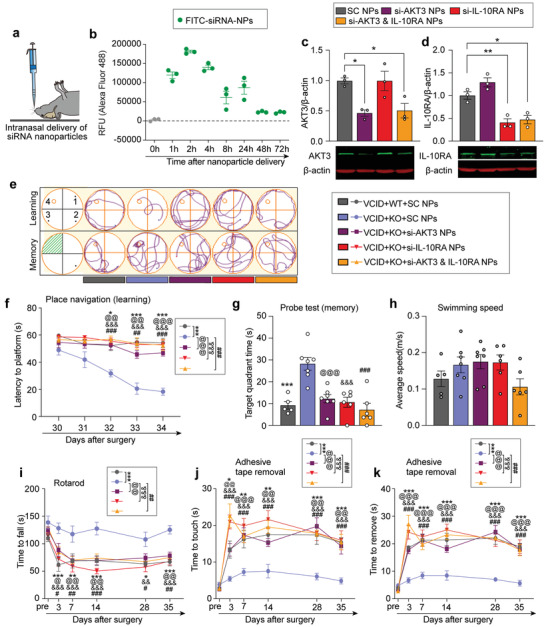
Knockdown of cerebral AKT3 and IL‐10RA partially abolishes long‐term neurobehavioral improvements in miR‐15a/16‐1 KO mice after VCID. a) Schematic diagram of intranasal siRNA‐loaded NP administration. b) Quantitative analysis of fluorescence intensity in mouse brains after intranasal delivery of FITC‐siRNA‐NPs (n = 3/group). c,d) Western blotting images and quantitative analysis showing cerebral AKT3 and IL‐10RA expression at 5 d after AKT3 and IL‐10RA siRNA‐NP delivery (n = 3/group, one‐way ANOVA & Bonferroni's test). Cognitive function was evaluated by the MWM test at 30–35 d of VCID in miR‐15a/16‐1 KO and WT mice with the treatment of AKT3 and IL‐10RA siRNA‐NPs. e) Swim paths in learning and memory phases during the MWM test. f) The latency to find the hidden platform in the place navigation phase (learning). g) The swim time in the target quadrant in the probe test (memory). h) Average swimming speed in the MWM test. Sensorimotor function was also evaluated in miR‐15a/16‐1 KO and WT mice with the treatment of AKT3 and IL‐10RA siRNA‐NPs by rotarod and adhesive tape removal tests at indicated VCID time points. i) The time to fall in the rotarod test. j) The time to touch and k) time to remove the tape in the adhesive tape removal test. Data are presented as mean ± SEM, n = 5‐7/group. ^*^, ^@^, ^&^, ^#^ *p* < 0.05; ^**^, ^@@^, ^&&^, ^##^ *p* < 0.01; and ^***^, ^@@@^, ^&&&^, ^###^ *p* < 0.001 versus VCID+KO+ SC NPs. Statistical analyses were performed by one‐way ANOVA and Bonferroni's post‐hoc test (c, d, g, h) and two‐way ANOVA with Bonferroni's test (f, i–k).

In order to evaluate whether inhibition of AKT3 and IL‐10RA in VCID brains reduces or reverses neurobehavioral improvements conferred by genetic deletion of miR‐15a/16‐1, siRNA‐loaded nanoparticles (NPs) were administered by intranasal delivery (**Figure** **8**a). As indicated in Figure 8b, fluorescein isothiocyanate (FITC) conjugated siRNA‐loaded NPs were intranasally administered to mice and then mice were sacrificed at 1, 2, 4, 8, 24, 48, and 72 h after NP delivery. The fluorescence intensity in the supernatant of mouse brain tissue was quantitatively assessed. As shown in Figure 8b, high fluorescence intensity was detected from 1 h to 72 h after NP delivery with a peak value at 2 h, indicating effective intranasal delivery of siRNA‐loaded NPs into mouse brains. Then, AKT3 or IL‐10RA siRNA‐loaded‐NPs (2µg siRNA in 12µL NPs/mouse) were intranasally delivered to the mice and the silencing efficiency of siRNA was examined at 5 days after delivery. As shown in Figure 8c, d and Figure S13, Supporting Information, the protein levels of AKT3 or IL‐10RA were significantly reduced in mouse brains 5 d after delivery.

Based on the above preliminary results, either scrambled control (SC), AKT3‐, or IL‐10RA siRNA‐loaded NPs were intranasally delivered to mice at ‐1, 5, 10, 15, 20, 25, and 30 d after VCID. Then these experimental mice were subjected to neurobehavioral testing as described above (Figure S14, Supporting Information). As shown in Figure 8e–h (MWM test), compared with the VCID+KO+SC NP group, siRNA silencing of AKT3 and/or IL‐10RA almost abolished the improved learning and memory function in miR‐15a/16‐1 KO mice 35 d after VCID, showing longer latency to find the platform in place navigation test (Figure 8E, F) and less swim time in the probe test (Figure 8e, g). There were no differences in average swimming speed among the 5 indicated experimental groups in the MWM test (Figure 8h). In parallel, during the 5‐day interval of NP delivery, we continuously monitored sensorimotor function at −1, 3, 7, 14, 28, and 35 d after VCID. Our results showed that siRNA silencing of AKT3 and/or IL‐10RA almost abolished the improved sensorimotor function in miR‐15a/16‐1 KO mice 35 d after VCID, showing less staying time on the rod in the rotarod test (Figure 8i) and more time to touch or remove the tape in the adhesive tape removal test (Figure 8j, k). There were no changes in body weight or mortality among experimental groups treated with siRNA‐loaded NPs (Figure S15, Supporting Information).

Taken together, our data indicated that suppression of miR‐15a/16‐1 downstream target genes, AKT3 and/or IL‐10RA, eliminates the neurobehavioral improvements conferred by genetic deletion of miR‐15a/16‐1 in VCID mice.

### 2.9 Intranasal Delivery of AKT3 and IL‐10RA siRNA‐Loaded Nanoparticles Partially Blocks the Reduction of White Matter and Grey Matter Injury in miR‐15a/16‐1 KO Mice after VCID

**Figure 9:**
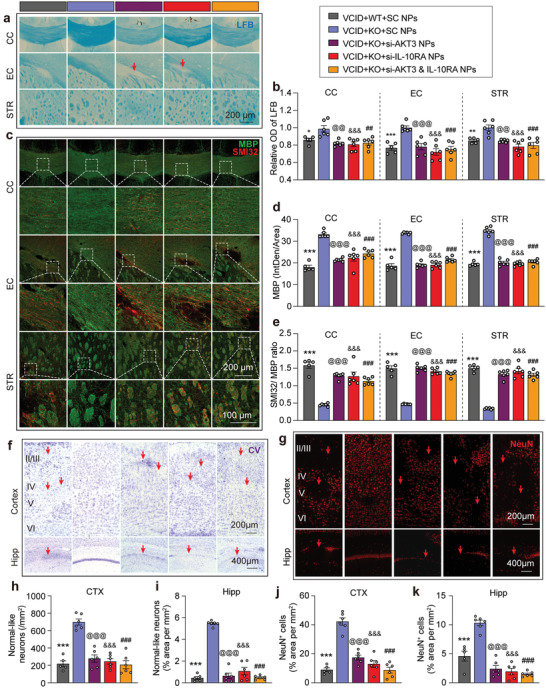
Knockdown of cerebral AKT3 and IL‐10RA partially blocks the reduction of WM and grey matter injury in miR‐15a/16‐1 KO mice after VCID. LFB histological staining and MBP/SMI32 immunofluorescence double staining were conducted in brains from miR‐15a/16‐1 KO and WT mice intranasally treated with AKT3 and IL‐10RA siRNA‐loaded‐NPs to detect myelin loss and axonal damage at 35 d after VCID. a) Representative images of LFB staining in the CC, EC, and STR areas. b) Quantitative analysis of relative OD values of LFB in the CC, EC, and STR (n = 5–6/group, one‐way ANOVA & Bonferroni's test). c) Representative images of MBP (green) co‐immunostained with SMI32 (red). d) Quantitative analysis of MBP mean fluorescence intensities and e) ratio of SMI32/MBP in the CC, EC, and STR (n = 5–6/group, one‐way ANOVA & Bonferroni's test). CV and NeuN staining were conducted in miR‐15a/16‐1 KO and WT mice intranasally treated with AKT3 and IL‐10RA siRNA‐loaded‐NPs to evaluate grey matter damage at 35 d after VCID. f,g) Representative images of CV and NeuN (red) staining in the CTX and Hipp regions. h,i) Quantitative analysis of normal‐like neurons in the CTX and Hipp areas. j,k) Quantitative analysis of NeuN‐positive neurons in the CTX and Hipp areas (n = 5–6/group, one‐way ANOVA & Bonferroni's test). Data are presented as mean ± SEM. ^*^, ^@^, ^&^, ^#^ *p* < 0.05; ^**^, ^@@^, ^&&^, ^##^ *p* < 0.01; and ^***^, ^@@@^, ^&&&^, ^###^ *p* < 0.001 versus VCID+KO+SC NPs group.

We next examined whether inhibition of AKT3 and/or IL‐10RA by intranasal delivery of siRNA‐loaded NPs affects white and grey matter protection conferred by genetic deletion of miR‐15a/16‐1. As shown in **Figure** **9**a, c, intranasal delivery of AKT3 and/or IL‐10RA‐siRNA‐loaded NPs caused severer WM injury, myelin loss, and axonal damage when compared with the VCID+KO+SC NP group, as further evidenced by quantitative analysis revealing decreased OD values in LFB stain (Figure 9b), decreased mean fluorescence intensity of MBP (Figure 9d), and the increased ratio of SMI32/MBP (Figure 9e) in the CC, EC, and striatum areas. In addition, the fluorescence intensity of SMI32 in the above brain regions was also significantly increased after AKT3 and/or IL‐10RA‐siRNA‐loaded‐NPs were delivered to miR‐15a/16‐1 KO mice after VCID (Figure S16a–d, Supporting Information). Further correlation analysis showed that MBP intensity in the CC, EC, and STR was positively related to cognitive function, while SMI32 intensity in the CC, EC, and STR is negatively related to cognitive function after siRNA‐loaded‐NP delivery (Figure S16e, Supporting Information). These results demonstrate the strong correlation of AKT3 and IL‐10RA with cognitive function after VCID.

Grey matter injury was also detected by CV and NeuN staining (Figure 9f, g) after intranasal administration of AKT3 and/or IL‐10RA‐siRNA‐loaded NPs. As shown in Figure 9f, g, fewer NeuN‐positive cells and less normal‐like neurons were found in the cerebral cortex and hippocampus in AKT3‐ and/or IL‐10RA siRNA NP‐treated miR‐15a/16‐1 KO mice compared with SC siRNA NP‐treated miR‐15a/16‐1 KO mice 35 d after VCID (Figure 9h–k), suggesting aggregated VCID‐derived grey matter injury in miR‐15a/16‐1 KO mice after siRNA silencing of cerebral AKT3 and IL‐10RA.

These data reveal that the protective effects of white/grey matter after VCID conferred by genetic deletion of miR‐15a/16‐1 depend on increased expression of AKT3 and IL‐10RA in the brain. Suppression of AKT3 and/or IL‐10RA blocks brain structural and functional protection conferred by genetic deletion of miR‐15a/16‐1 in VCID mice.

### 2.10 Intranasal Delivery of AKT3 and IL‐10RA siRNA‐Loaded Nanoparticles Increases Astrocytic NF‐*κ*B p65 Activation in miR‐15a/16‐1 KO Mice after VCID

To further explore the downstream mechanism of the miR‐15a/16‐1/IL‐10RA/AKT3 regulatory axis in VCID. We examined NF‐*κ*B p65 expression in astrocytes by co‐immunostaining the NF‐*κ*B p65 subunit with GFAP. As shown in Figure S17, Supporting Information, compared with the VCID+KO+SC NPs group, siRNA silencing of AKT3 and/or IL‐10RA significantly increased astrocytic NF‐*κ*B p65 expression in miR‐15a/16‐1 KO mice 35 d after VCID. This result suggests that downregulation of IL‐10RA and/or AKT3 in astrocytes increases the pro‐inflammatory response via activation of NF‐*κ*B p65, implying that NF‐*κ*B may be one of the downstream targets of the miR‐15a/16‐1/IL‐10RA/AKT3 regulatory axis in VCID.

## 3 Discussion

In this study, we found that genetic deficiency of miR‐15a/16‐1 improves long‐term sensorimotor and cognitive declines in VCID mice. Genetic deletion of miR‐15a/16‐1 also preserves WM and grey matter integrity in mice after VCID. These brain‐protective effects mediated by in vivo loss‐of‐miR‐15a/16‐1 function depend on the reduction of astrocytic activation and preservation of anti‐inflammatory mediators, AKT3 and IL‐10RA in both white and grey matter after VCID. Furthermore, the suppression of AKT3 and/or IL‐10RA by siRNA‐loaded NPs partially abolished the improvement of neurobehavioral function and reduction of WM and grey matter injury in miR‐15a/16‐1 KO mice after VCID. Our results reveal the important role of the miR‐15a/16‐1/IL‐10RA/AKT3 axis in regulating the pathological progression of VCID.

Unlike a sudden reduction of regional CBF in ischemic stroke, vascular dementia is associated with a moderate and persistent decrease in CBF. Neuroimaging studies have revealed remarkable CBF reduction in the cerebral cortex, especially in the frontal cortex of patients with VCID, which is closely associated with increased subcortical WM injury.^[^ ^18^ ^]^ Besides, patients with mild cognitive impairment and reduced CBF patterns are vulnerable to dementia.^[^ ^19^ ^]^ In the past years, various experimental VCID rodent models have been established to recapitulate CCH‐derived brain damage in VCID.^[^ ^20^ ^]^ Hattori et al. reported a novel vascular cognitive impairment mouse model, ACAS, which can simulate VCID patients.^[^ ^6b^ ^]^ Unlike the rapid reduction of CBF in other experimental VCID models such as bilateral common carotid artery stenosis (BCAS) and bilateral common carotid artery occlusion (2‐VO), the mouse ACAS model is characterized by gradual and persistent reduction of CBF.^[^ ^6^, ^21^ ^]^ In the present study, we observed prolonged CBF reduction in WT mice subjected to ACAS, confirming the previous report by Hattori's group. Of note, we revealed that genetic deficiency of miR‐15a/16‐1 effectively reduced long‐term CBF decline, which may be associated with miR‐15a/16‐1 silencing‐triggered enhancement of VEGFA and FGF2 and subsequent cerebral pro‐angiogenic activity in VCID mice. This finding is consistent with our previous report that endothelium‐targeted miR‐15a/16‐1 genetic deletion improves CBF recovery and promotes peri‐infarcted brain angiogenesis and long‐term neurological recovery in mice after focal cerebral ischemia.^[^ ^16a^ ^]^ Thus, the protective effects of miR‐15a/16‐1 genetic deletion on CBF recovery after ACAS may contribute to delayed pathological progression in VCID.

Our previous data have shown that endothelium‐targeted miR‐15a/16‐1 genetic deletion can reduce long‐term sensorimotor and cognitive impairments in mice after cerebral ischemia.^[^ ^16a^ ^]^ Consistent with our previous findings in a mouse stroke model, we observed obvious sensorimotor deficits and cognitive impairments in the ACAS‐based mouse VCID model. Genetic deletion of miR‐15a/16‐1 improves long‐term motor coordination and balance function as well as learning and memory capacities after VCID, suggesting that inhibiting miR‐15/16‐1 activity may represent a promising therapeutic approach to lessening cognitive decline or dementia in VCID.

WM injury is a classic feature in patients with VCID, experimental VCID models, and other dementias.^[^ ^1^, ^5^, ^22^ ^]^ As a non‐invasive clinical approach, DTI has been widely applied to detect WM injury in the human brain. Previous studies have reported that the FA value, an indicator of WM damage in DTI, was decreased in TBI, Alzheimer's disease, and vascular dementia.^[^ ^23^ ^]^ MicroRNAs can participate in the development and regulation of WM integrity.^[^ ^24^ ^]^ For example, altered microRNA levels have been observed during the onset of multiple sclerosis (MS, a demyelinating disease) and Alzheimer's disease.^[^ ^25^ ^]^ Endothelial‐cell targeted deletion of miR‐126 accelerated WM lesions after VCID.^[^ ^14b^ ^]^ Silencing of miR‐125a‐3p promoted WM demyelination.^[^ ^26^ ^]^ Although miR‐15a/16‐1 genetic deletion reduced post‐stroke neuronal loss and brain infarct size,^[^ ^15^ ^]^ its effect on WM lesions is still unclear.

The present study is the first to elucidate that genetic deletion of miR‐15a/16‐1 prevented decline of the FA value and fiber intensity in VCID brains. Subsequent histological and immunohistochemical studies further confirmed these results from DTI. Genetic deficiency of miR‐15a/16‐1 significantly increased the OD value in LFB staining and MBP fluorescence intensity, and decreased SMI32 fluorescence intensity in mouse brains after VCID in comparison with WT controls. In addition, ultrastructural imaging of TEM also showed that miR‐15a/16‐1 KO mice exhibited thicker myelin sheaths, smaller numbers of abnormal myelin, and lower g‐ratios after 35 d of VCID compared with WT controls. Taken together, these data indicate that genetic deficiency of miR‐15a/16‐1 confers a significant WM protective role in VCID brains.

Microinfarcts and lacunar infarcts are independent predictors of cognitive dysfunction and are commonly associated with vascular dementia and other dementia‐related diseases.^[^ ^9^ ^]^ Patients with neuronal apoptosis in neocortical layers III and V are more vulnerable to dementia.^[^ ^1a^ ^]^ In addition, hippocampal neuronal density in the CA1, but not CA2 subfield was correlated to cognitive/memory dysfunction in vascular dementia.^[^ ^27^ ^]^ Our previous studies showed that general miR‐15a/16‐1 genetic deletion reduced brain tissue loss and brain infarction in stroke mice in comparison with WT controls. Another study also demonstrated that the miR‐15a mimic aggregated OGD‐induced neuronal injury in vitro.^[^ ^28^ ^]^ In this study, neuronal loss in multiple layers of the cerebral cortex and hippocampal CA1 territory was detected in mouse VCID brains, consistent with the previous ACAS study.^[^ ^6^, ^29^ ^]^ In addition, we observed that genetic deletion of miR‐15a/16‐1 significantly increased the expression of synaptic plasticity‐related proteins PSD‐95 and synaptophysin in VCID brains, suggesting that miR‐15a/16‐1 genetic deficiency may improve cognition in VCID through the regulation of synaptic plasticity. We also found that VCID caused a significant reduction in the hippocampal volume in WT mice. Of note, miR‐15a/16‐1 genetic deletion not only mitigated neuronal loss in the cerebral cortex and hippocampal CA1 subfield but also prevented atrophy of the hippocampus. It is the first report to document that genetic deficiency of miR‐15a/16‐1 confers a significant neuroprotective role in VCID brains.

Chronic neuroinflammation is known as a critical factor in accelerating neuropathological progression, such as WM and grey matter lesions in VCID.^[^ ^9^ ^]^ Poh and colleagues demonstrated that astrocytes significantly increased in the striatum 1d after the onset of VCID and reached a maximal level at 7 d after VCID.^[^ ^8a^ ^]^ Astrocytes were still maintained at high levels in the cerebral cortex and EC regions at 28–42 d after the onset of VCID.^[^ ^30^ ^]^ Activated astrocytes triggered the NF‐kB inflammatory signaling pathway and increased inflammasomes that further promote the pathological progression of VCID.^[^ ^8^, ^30^ ^]^ In the current study, we found significantly increased numbers of astrocytes in both WM and grey matter (CC, EC, STR, and CTX) 3 d after VCID in WT mice. Encouragingly, genetic deletion of miR‐15a/16‐1 significantly reduced astrocyte activation in WM and grey matter, indicating that inhibition of miR‐15a/16‐1 activity provides anti‐proinflammatory effects on VCID brains. AKT3 is a brain‐enhanced isoform of the AKT family and represents 50% of the total AKT in the brain.^[^ ^17b^ ^]^ Li et al. reported that downregulation of the AKT pathway increased S100A10 (a marker for the A2 astrocyte phenotype) and decreased C3 (a marker for the A1 astrocyte phenotype) in astrocytes.^[^ ^31^ ^]^ As the upstream receptor of AKT3, IL‐10RA also plays a vital role in inhibiting neuroinflammation. O'Neil et al. showed that genetic deficiency of IL‐10RA in astrocytes caused exaggerated sickness behavior and a prolonged neuroinflammatory response to peripheral LPS by decreasing astrocytic expression of transforming growth factor (TGF)‐*β*, an anti‐inflammatory cytokine.^[^ ^32^ ^]^ These studies indicate that the IL‐10RA/AKT3 signaling pathway can suppress astrocyte activation via multiple targets. In the present study, we reported that the reduction of IL‐10RA and AKT3 mRNA and protein in VCID brains were reversed by the genetic deletion of miR‐15a/16‐1 in mice. We also observed the NF‐*κ*B p65 decline in astrocytes after 35 d of VCID in miR‐15a/16‐1 KO mice. Moreover, siRNA silencing of AKT3 and/or IL‐10RA significantly increased astrocytic NF‐*κ*B p65 expression in miR‐15a/16‐1 KO mice 35 d after VCID. These results suggested that downregulation of IL‐10RA and/or AKT3 in astrocytes increases the pro‐inflammatory response via activation of NF‐*κ*B p65, implying that NF‐*κ*B may be one of the downstream targets of the miR‐15a/16‐1/IL‐10RA/AKT3 axis in VCID. Taken together, our findings suggest a vital regulatory role of the miR‐15a/16‐1/IL‐10RA/AKT3 axis in astrocyte activation and neuroinflammation after VCID.

To elucidate the underlying regulatory mechanisms of miR‐15a/16‐1 in the regulation of vascular brain damage and cognitive decline in VCID brains, we conducted bioinformatics analysis of mouse miR‐15a/16‐1 target genes that are related to inflammation, revealing AKT3 as one of the major candidates. DuBois et al. reported that mice with AKT3 genetic deletion exhibited severer demyelination and increased inflammation in the spinal cord and brain of the mouse model of autoimmune encephalomyelitis (EAE) when compared with WT controls.^[^ ^17b^ ^]^ Meanwhile, AKT3 is involved in the regulation of neurotransmission and synaptic plasticity.^[^ ^33^ ^]^ Inhibition of AKT3 in mouse embryonic stem cells (ESCs) impacts cell survival and proliferation.^[^ ^34^ ^]^ Besides, Liby et al. demonstrated that AKT3 regulates VEGF secretion and angiogenesis in ovarian cancer cells.^[^ ^35^ ^]^ These data suggest that AKT3 is highly involved in regulating the anti‐inflammatory response, cell proliferation, cell survival, neuronal homeostasis, angiogenesis, and WM integrity. We have previously reported that miR‐15a/16‐1 can negatively regulate the expression of Bcl‐2, claudin 5, VEGFA, FGF2, VEGFR2, and FGFR1 to modulate post‐stroke cerebrovascular injury and remodeling.^[^ ^15^, ^16^ ^]^ We have shown that genetic deletion or pharmacological inhibition of miR‐15a/16‐1 activity provides brain protection and repair in experimental stroke by inhibiting apoptosis, maintaining blood‐brain barrier integrity, and promoting cerebral angiogenesis. Spinetti et al. demonstrated that miR‐15a/16‐1 downregulated the expression of AKT3 by directly binding to the 3’‐UTR of AKT3 mRNA in proangiogenic circulating cells (PACs) after critical limb ischemia, while inhibition of miR‐15a/16‐1 increased the expression of AKT3 and pAKT in PACs.^[^ ^36^ ^]^ In the present study, we showed that the expression of AKT3 was decreased in vulnerable brain regions 3 d after VCID. We also confirmed that miR‐15a/16‐1 directly binds to the 3’‐UTR of AKT3 mRNA and translationally inhibits the expression of AKT3 in HEK 293 cells. Moreover, genetic deletion of miR‐15a/16‐1 increased the mRNA and protein levels of AKT3 and phosphorylated AKT in both the CC/EC and cerebral cortex after VCID when compared with WT mice. Furthermore, miR‐15a/16‐1 negative regulation of AKT3 was also confirmed by immunofluorescence staining, showing that AKT3 is expressed in both WM and grey matter in miR‐15a/16‐1 KO and WT mice, and genetic deletion of miR‐15a/16‐1 prevented the decline of AKT3 in these areas after VCID. Our data indicate that miR‐15a/16‐1 regulates vascular brain damage and cognitive decline in VCID brains via translational inhibition of its downstream target gene, AKT3.

In parallel, we also focused on and evaluated the role of another potential downstream target gene of miR‐15a/16‐1, interleukin 10 receptor alpha (IL‐10RA) in VCID brains. A previous study showed that miR‐15a regulates the development and progression of melanoma by post‐transcriptionally inhibiting IL‐10RA in G361 melanoma cells.^[^ ^37^ ^]^ IL‐10 can protect neurons and reduce pro‐inflammatory responses in vitro after OGD damage through PI3K/AKT and STAT‐3 pathways.^[^ ^38^ ^]^ Ledeboer et al. reported that IL‐10 was elevated but IL‐10RA was decreased after oxygen‐glucose deprivation (OGD) in cultured rat glial cells.^[^ ^39^ ^]^ Besides, cerebral expression of IL‐10RA was reduced 3 d after permanent ischemic stroke.^[^ ^40^ ^]^ Puntambekar et al. demonstrated that IL‐10 genetic deletion in mice induced severer WM lesions and demyelination in a hepatitis virus‐induced demyelination model.^[^ ^17a^ ^]^ Moreover, inhibition of IL‐10/IL‐10R has been shown to block angiogenesis and migration in human umbilical vein endothelial cells.^[^ ^41^ ^]^ Consistent with the previous report, we also confirmed a direct regulatory function of miR‐15a/16‐1 on IL‐10RA in HEK 293 cells. We further demonstrated the mRNA and protein levels of IL‐10RA were enhanced in the CC/EC and cerebral cortex from VCID mice with genetic deletion of miR‐15a/16‐1. Furthermore, miR‐15a/16‐1 negative regulation of IL‐10RA was also confirmed by immunofluorescence staining, showing that IL‐10RA is expressed in both WM and grey matter in miR‐15a/16‐1 KO and WT mice, and genetic deletion of miR‐15a/16‐1 prevented the decline of IL‐10RA in these areas 3 d after VCID. Our data indicate that miR‐15a/16‐1 regulates vascular brain damage and cognitive decline in VCID brains via translational inhibition of its downstream target gene, IL‐10RA.

Intranasal administration is a non‐invasive approach for drug delivery into the central nervous system which may bypass the BBB.^[^ ^42^ ^]^ This local administration through the intranasal route is considered more effective and specific to the brain than other systemic delivery approaches such as intravenous and intraperitoneal injections. NPs can facilitate drug transport across the BBB and protect against and delay drug/chemical degradation. Sanchez‐Ramos et al. demonstrated that NP‐siRNA delivery induced more than 50% GFP silencing efficiency in different brain regions 48h after administration, and MRI tracking showed NPs were distributed to whole brain regions after intranasal delivery.^[^ ^43^ ^]^ Several other studies have explored the potential for intranasal siRNA‐loaded NP delivery for the treatment of CNS diseases, such as Alzheimer's disease, Parkinson's disease, and stroke.^[^ ^43^, ^44^ ^]^ For example, intranasal delivery of iNOS siRNA‐loaded NPs protects mice from ischemic brain injury.^[^ ^44c^ ^]^ Intranasal NP delivery of Beclin1‐siRNA achieves HIV attenuation in the brain and may have therapeutic effects on Alzheimer's disease.^[^ ^45^ ^]^ In our present study, the expression of AKT and/or IL‐10RA was significantly inhibited by intranasal delivery of siRNA‐loaded NPs in miR‐15a/16‐1 KO mice after VCID. Of importance, genetic deficiency of miR‐15a/16‐1‐derived brain protection and neurobehavioral improvements are almost abolished by AKT3 and/or IL‐10RA silencing via intranasal delivery of siRNA‐loaded NPs, showing aggravated cognitive dysfunction, sensorimotor deficits, and WM and grey matter injury in VCID brains. These data are the first to provide convincing evidence for the proof‐of‐concept for the miR‐15a/16‐1/IL‐10RA/AKT3 axis in the regulation of VCID.

In conclusion, we reported for the first time that genetic deletion of miR‐15a/16‐1 preserves AKT3 and IL‐10RA activity in both WM and grey matter after VCID, which promotes the recovery of sensorimotor and cognitive functions and protects against WM and grey matter injury after VCID. Therefore, the findings of this study elucidate the regulatory role of the miR‐15a/16‐1/IL‐10RA/AKT3 axis in the intervention of VCID.

## 4 Experimental Section

### Animals and Experimental Design

All procedures using laboratory animals were approved by the University of Pittsburgh Institutional Animal Care and Use Committee (IACUC) (18073370) and performed in accordance with the National Institutes of Health Guide for the Care and Use of Laboratory Animals. Mice were housed in groups of four per cage in a temperature‐ and humidity‐controlled animal facility with a 12‐h light‐dark cycle. Food and water were available ad libitum. All efforts were made to minimize animal suffering and the number of animals euthanized.

Male homozygous microRNA‐15a/16‐1 KO mice were kindly provided by Dr. Riccardo Dalla‐Favera.^[^ ^46^ ^]^ Male littermate WT mice were used for controls (8–12 w, 23–30 g). Both miR‐15a/16‐1 KO and WT mice were randomly assigned to either the sham or VCID group by a lottery box. All outcomes were performed by independent investigators blinded to procedures and mouse types.

### Mouse Model of Vascular Cognitive Impairment and Dementia (VCID)

Experimental VCID was induced by ACAS as described previously.^[^ ^6b^ ^]^ Briefly, mice were anesthetized with 1.5% isoflurane in a mixture of 25% O_2_ and 74% N_2_O during surgical procedures. Then, a microcoil (wire diameter 0.08 mm; inside diameter 0.18 mm; length 2.5 mm; Wuxi Samini Spring Co.) was vexed to the right CCA and the AC (inner diameter = 0.5 mm; Tokyo Instruments) was placed in the left CCA. Rectal temperature was controlled at 37.0 ± 0.5 ℃ and heart rate was monitored during surgery.

### Cognitive and Sensorimotor Neurobehavioral Tests

A series of neurobehavioral tests were carried out before and up to 35 days after ACAS to evaluate cognitive and sensorimotor deficits by an individual blinded to experimental groups.

### Morris water maze test

Long‐term spatial learning and reference memory were evaluated by the MWM test at 31–35 days after ACAS as described previously.^[^ ^16^, ^22^ ^]^ Before surgery, mice were placed into a water tank to explore freely for 90 s. Mice that were motionless while exploring the water tank were excluded from formal testing. In the place navigation (learning) phase, a circular platform was immersed below the water surface and placed in the middle of the 4th quadrant (target quadrant). Mice were released to the pool from the 1st, 2nd, and 3rd quadrants in a random order each day and the times to find the platform in each trial were recorded. The probe test (memory) on the 6th day was conducted by removing the hidden platform. Mice were allowed to explore for 60 s and the swimming speed and time expended in the target quadrant were recorded. ANYMAZE software and a video recording system were used for recording MWM tests.

### Novel objective recognition test

To evaluate long‐term memory decline, the NOR test was performed as described in previous publications with minor modifications.^[^ ^47^ ^]^ First, in the habituation phase, mice were placed in the open‐field arena to freely explore without objects for 5 mins. Then, in the familiarization phase, mice were placed in the open‐field arena with two identical sample objects (old 1 + old 2) to freely explore for 10 mins. The mice were released against the objects and facing the wall to prevent coercion. After 1 h of retention interval in its housing cage, in the test phase, mice were released to the open‐field arena again with two objects, one was identical to the sample object and the other was a novel object (old 1 + novel) for another 10 mins of exploration. The exploration time in the test phase for old and novel objects was recorded using ANYMAZE software and a video recording system. The discrimination index was calculated by the following formula: Discrimination index = (T_novel_−T_old1_)/(T_novel_+T_old1_).

### Rotarod test

Long‐term sensorimotor function was evaluated by the rotarod test before and at 3, 7, 14, 28, and 35 days of ACAS as described previously.^[^ ^16a^ ^]^ Briefly, experimental mice were placed into a rotating drum (IITC Life Science Inc.) accelerating from 5 rpm to 40 rpm within 5 min. Three trials per day were performed for 3 consecutive days before and up to 35 days of surgery with a 5‐min interval between each trial. The mean time on the rod (latency to fall) of three trials 1 day before surgery (baseline) and selected timepoints after surgery were recorded.

### Adhesive tape removal test

Forepaw sensorimotor functions were evaluated by the adhesive tape removal test as described previously.^[^ ^48^ ^]^ Experimental mice were placed into a transparent Plexiglas cylinder (30 cm tall by 20 cm diameter) for a 60 s habituation period before testing. Then, a piece of adhesive tape (0.3×0.4 cm) was placed on the right hairless part of the forepaws. The time to when the adhesive tape was touched (time to touch) and completely removed (time to remove) from forepaw was recorded. Three trials per day were conducted 1 day before surgery and also selected timepoints after surgery. The maximum time between touch and removal was recorded as 120 s if the mouse did not touch or remove the adhesive tape.

### Corner test

Sensorimotor function was evaluated by the corner test as described in a previous publication^[^ ^5c^ ^]^ with minor modifications. Briefly, a small corner with a 30° angle was formed by two Plexiglas boards (30 cm x 20 cm x 1 cm). Experimental mice were placed halfway to the corner for 10 times per day before and after up to 35 days of surgery. Upon entering the corner, the mice reared forward and upward before making a U‐turn. The frequency of left turns and right turns were recorded. Turning movements that were not part of a rearing movement were not recorded.

### Laser Speckle Imaging

Changes in CBF were monitored by a laser speckle imager (Pericam PSI, PERIMED, NV, USA) before and after 3, 7, 14, 28, and 35 days of surgery.^[^ ^49^ ^]^ Briefly, experimental mice were anesthetized with 1.5–3% isoflurane and placed on a 37 °C heating pad in a prone position. A midline scalp incision was made, and the skin was separated with two forceps. The laser speckle imager was placed 10 cm above the exposed skull surface. Five images per the above timepoints were captured for each mouse. Reginal CBF was calculated and analyzed with PeriCam PSI software. The relative CBF was calculated as the mean value of five images per mouse in both the AC side and microcoil side, which were then normalized to the mean values of pre‐surgery CBF baseline levels for each animal.

### Diffusion Tensor Imaging (DTI)

Small animal in vitro DTI was performed to evaluate WM injury and brain atrophy as described previously.^[^ ^23a,b^ ^]^ After 35 days of ACAS surgery, mice were sacrificed and scanned using a vertical‐bore 11.7 Tesla/89mm Bruker AVANCE AV3 HD MicroImaging System equipped with a Micro2.5 gradient insert and a 20‐mm quadrature RF probe with ParaVision 6.0.1 (Bruker Biospin, Billerica MA). DTI data were collected using a multi‐slice spin‐echo sequence with 5 A0 images and 30 non‐colinear diffusion‐weighted images with the following parameter: TE/TR 22/2800 ms, 2 averages, 160 × 160 matrix, 16 × 16‐mm FOV, 25 slices, 0.5 mm slice thickness, b‐value = 3000 s mm^−2^, and Δ/*δ* = 11.0/5.0 ms. The DTI directionally encoded color (DEC) maps and FA maps were generated with DSI Studio software (http://dsistudio.labsolver.org/). The coincident ROI in the CC and EC of each mouse was drawn, and the FA value was recorded. Cortical and hippocampal volumes were measured by combing the ROI of all scan levels.

### Transmission Electron Microscopy (TEM)

To observe the ultrastructure of the myelin sheath in the CC/EC arena, TEM was performed according to previous reports.^[^ ^5c^ ^]^ In brief, mice were sacrificed and perfused after 35 days of ACAS surgery. The CC/EC region was cut into 1 mm^3^ for preparing ultra‐thin sections with a Reichert Ultra‐microtome. Micrographs were captured with a Philips CM120 electron microscope (F.E.I., Hillsboro, OR). An average of 100 axons were measured by Image J software in each group. Then, myelin thickness, abnormal axons, and g‐ratios of individual fibers in relation to respective axon diameters (presented as scatter plots) were calculated and analyzed.

### Immunofluorescence (IF) Staining

IF staining was performed as previously described.^[^ ^16b^ ^]^ Briefly, mice were deeply anesthetized and transcardially perfused with 0.9% NaCl and then with 4% paraformaldehyde (PFA), and then brains were harvested and post‐fixed in 4% PFA overnight at 4 °C and immersed into 30% sucrose in 0.1 m PB for two days. By using a microtome (ThermoFisher HM450), brains were cut into 25‐µm coronal sections. The sections were stored at −20 °C until use. Sections were washed 3 times x 5 min with PBS, 1 time x 20 min with 1% PBST (1% Triton‐X 100 in PBS), and 2 times x 5 min with 0.3% PBST. The free‐floating sections were then blocked with 5% normal goat serum in 0.3% PBST for 1 h at room temperature. Then, brain sections were incubated with primary antibodies (diluted in 0.3% PBST) and followed by secondary antibodies accordingly. The primary antibodies used in this study and the corresponding dilution factor and vendor information are listed in Table S1, Supporting Information. Images were captured by a confocal microscope (FluoView FV1000, Olympus, Japan; A1R, Nikon, Japan). Mean fluorescence intensities and cell numbers were processed with Image J software. Three randomly selected microscopic fields on 3 consecutive sections in the cortex, CC, EC, striatum, and hippocampus were analyzed for each brain. Imaris software (Bitplane, Belfast, United Kingdom) was used to reconstruct 3D images of AKT3/IL‐10R double immunostained with NeuN, GFAP, Iba‐1, CD31, and APC as described previously.^[^ ^16a^ ^]^ The astrocytic skeleton was reconstructed by Imaris software based on GFAP immunostaining. Astrocytic branch area, branch length, branch points, and branch terminal points were analyzed with Imaris software as well. Surface area, capillary number, branch points, and vascular length of brain microvessels were analyzed by ImageJ software based on CD31 immunostaining.

### Crystal Violet (CV) Staining

CV staining was conducted to observe neuronal loss.^[^ ^8a^ ^]^ Briefly, brain sections were deionized and immersed into CV solution followed by distilled water, 70% alcohol, 95% alcohol, 100% alcohol, and xylene. Three randomly selected microscopic fields on 3 consecutive sections in the cortex and hippocampus were used for counting normal‐like neurons in each brain. CV staining of 16 coronal brain sections (bregma: +2.8 mm to −4.7 mm, 0.5 mm interval) was used for the assessment of cerebral cortical and hippocampal volume.

### Luxol Fast Blue (LFB) Staining

LFB staining was conducted to evaluate WM demyelination changes.^[^ ^8^, ^50^ ^]^ Briefly, brain sections were immersed into LFB solution, decolored with 70% alcohol and 0.05% Li_2_CO_3_, and dehydrated with graded ethanol. Images were taken with an EVOS microscope. Three randomly selected microscopic fields on 3 consecutive sections were used for calculating the relative OD values in the CC, EC, and striatum in each brain with Image J software.

### Western Blot

Western blotting was performed as previously described.^[^ ^16a^ ^]^ Total protein was extracted from the cerebral cortex, separated by 4–15% SDS‐PAGE (Bio‐rad) gel, and transferred to 0.45µm polyvinylidene difluoride (PVDF) membranes. After blocking with 5% non‐fat dried milk in 0.1% TBST buffer, membranes were incubated with pAKT3 (Ser473), AKT3, IL‐10RA, VEGFA, FGF2, PSD‐95, synaptophysin, or *β*‐actin primary antibody and incubated at 4 ℃ overnight in a shaker. After washing with 0.1% TBST 3 times each for 5 min, PVDF membranes were incubated with secondary antibodies for 1h at room temperature. Then, blots were washed three times with 0.1% TBST and protein levels were detected by an Odyssey LI‐COR scanner and analyzed by Image J software. The pAKT3 (Ser473), AKT3, IL‐10RA, VEGFA, FGF2, PSD‐95, and synaptophysin expression levels were normalized to *β*‐actin. Primary antibody dilution factors and vendor information for western blotting are listed in Table S1, Supporting Information.

### Quantitative PCR

Total RNA was extracted from the cerebral cortex of mouse brains by using Trizol (Invitrogen). Quantitative real‐time reverse transcriptase‐polymerase chain reaction (RT‐PCR) was carried out by using an iScript cDNA Synthesis Kit, iTaq Universal SYBR Green Supermix (Bio‐Rad, Hercules, CA, USA), and a Bio‐Rad CFX Connect Thermocycler, according to the published protocols.^[^ ^16^ ^]^ Specific primers for AKT3, IL‐10RA, and cyclophilin were used for the PCR reaction. The relative mRNA expression was normalized to cyclophilin RNA levels. PCR experiments were repeated 3 times, each using separate mouse brain samples. The detailed primer sequences used in this study are listed in Table S2, Supporting Information.

### Cell Culture and Dual‐Luciferase Reporter Assay

Dual‐luciferase reporter assays were conducted as previously described.^[^ ^16^, ^49^ ^]^ The mouse mIL‐10RA 3’‐UTR dual‐luciferase reporter plasmid (pEZX‐MT01‐mIL‐10RA 3’‐UTR) and mutated plasmid (pEZX‐MT01‐mIL‐10RA 3’‐UTR mutated), and the mouse mAKT3 3’‐UTR dual‐luciferase reporter plasmid (pEZX‐MT01‐mAKT3 3’‐UTR) and mutated plasmid (pEZX‐MT01‐mAKT3 3’‐UTR mutated) were directly purchased from GeneCopoeia. The four plasmid DNAs were transfected in *E. coli* bacteria on Kanamycin selection Luria Broth (LB) agar plates. The sequences of the plasmid DNAs extracted from bacterial colonies were validated by enzyme digestion and agarose electrophoresis. The concentration of plasmid DNA was measured with a NanoDrop 2000 Spectrophotometer (ThermoFisher Scientific, USA). The HEK 293 cell line was cultured with DMEM medium (with 10% FBS, without antibiotics) and seeded onto 24‐well plates. On the next day, when HEK 293 cells reached 30% confluency, cells were infected with lentiviruses for knockdown or overexpression of miR‐15a/16‐1. At 72 h after lentivirus infection, cells were transfected with either wild‐type or mutated mIL‐10RA or mAKT3 plasmids mentioned above by the Lipofectamine 2000 transfection reagent (Invitrogen, USA) and Opti‐MEM medium for 6 h. After 6 h of transfection, cells were subjected to a medium change to 10% FBS DMEM medium and cultured for another 24 h. Cells were harvested with Passive Lysis Buffer (PLB). Dual‐Luciferase assays (Promega) and a SpectraMax i3x microplate reader (Molecular Device, USA) were utilized to measure the luciferase activity of Firefly and Renilla. The relative Firefly luciferase activity was normalized to Renilla luciferase activity. Four individual experiments were conducted for obtaining the average luciferase activity.

### Nanoparticle Preparation and Intranasal Administration

SiRNA NPs were prepared as described previously^[^ ^51^ ^]^ with slight modifications. Briefly, siRNA in PBS was mixed with a cationic polymer at an N/P ratio of 10/1. The polymer/siRNA complex was then mixed with CS/CS‐PEG to obtain CS‐coated siRNA NPs. The size of the NPs was around ≈110 nm with a surface charge close to neutral. Mice were placed in a supine position on an electric water heating pad (37 °C) and anesthetized with 1.5% isoflurane in a mixture of 25% O_2_ and 74% N_2_O during administration processes. Before administration in VCID mice in formal experiments, FITC‐siRNA‐NPs were designed and intranasally delivered to the mice. After 1, 2, 4, 8, 24, 48, and 72 h of delivery, whole brain tissue was collected and sonicated in 500 µL 1% TBST. Then, the mixed suspension was centrifuged at 15 000 rpm for 3 min. After centrifugation, the supernatant (200 µL) was pipetted to a 96‐well plate, and the fluorescence intensity was measured with a plate reader. Three mice per time point were used to study brain penetrability of synthetic NPs.

A total of 12 or 24 µL CS‐coated siRNA NPs (2ug siRNA) were alternately dripped into both nasal cavities of each mouse with a pipette. For the VCID+WT+SC‐siRNA‐NPs group, VCID+miR‐15a/16‐1 KO+SC‐siRNA‐NPs group, VCID+miR‐15a/16‐1 KO+AKT3‐siRNA‐NPs, and VCID+miR‐15a/16‐1 KO +IL‐10RA‐siRNA‐NPs group, 12 µL of NPs were delivered to mice. For the VCID+miR‐15a/16‐1 KO+AKT3&IL‐10RA‐siRNA‐NPs group, 12 µL of AKT3‐siRNA‐NPs and 12 µL of IL‐10RA‐siRNA‐NPs were delivered to the mice (5 min interval). Mice were subjected to VCID the following day. After VCID, mice received intranasal administration of different NPs according to their experimental groups at 5, 10, 15, 20, 25, and 30 d of VCID. Respiration and body temperature were monitored during drug administration.

### Statistical Analyses

The normality of all data in this study was evaluated with the Shapiro–Wilk test. All data in this study were expressed as mean ± SEM with dots and analyzed with GraphPad Prism 9 (GraphPad software, CA, USA). For data that meets with the Shapiro–Wilk normality test and Brown–Forsythe homogeneity of variance test of multiple‐group comparison, one‐way or two‐way ANOVA was used followed by Bonferroni post‐hoc tests. Welch ANOVA and Dunnett T3 post‐hoc tests were used when the variances were heterogeneous. The Kruskal Wallis test was used when data distribution did not meet normal Gaussian distribution. Two‐tailed t‐test was used for two‐group comparison. Two‐tailed Pearson correlation analyses were employed for correlation analysis. A *p*≤0.05 was considered statistically significant.

## Conflict of Interest

The authors declare no conflict of interest.

## Author Contributions

Conceptualization: K.J.Y.; Methodology: C.Z., Y.X., P.S., Y.C., Y.H., S.L., L.F., and T.K.H.; Investigation: C.Z.; Visualization: P.S.; Supervision: K.J.Y.; Writing – original draft: C.Z.; Writing – review & editing: C.Z., P.S., K.J.Y., Y.C., T.K.H., S.L., and M.H.H.

## Supporting information

Supporting Information

## Data Availability

The data that support the findings of this study are available in the supplementary material of this article.
