# Supplementary material for: Genetic Deficiency of MicroRNA‐15a/16‐1 Confers Resistance to Neuropathological Damage and Cognitive Dysfunction in Experimental Vascular Cognitive Impairment and Dementia
Source: Adv Sci (Weinh). 2022 Apr 11;9(17):2104986. doi: 10.1002/advs.202104986 (PMC9189640; doi:10.1002/advs.202104986)
Supplement: Supplementary file 1 — Supporting Information [file ADVS-9-2104986-s001.pdf]

ADVANCED SCIENCE
Open Access

Supporting Information

for *Adv. Sci.*, DOI 10.1002/advs.202104986

Genetic Deficiency of MicroRNA-15a/16-1 Confers Resistance to Neuropathological Damage and Cognitive Dysfunction in Experimental Vascular Cognitive Impairment and Dementia

*Chao Zhou, Ping Sun, Yang Xu, Yuang Chen, Yixian Huang, Milton H. Hamblin, Lesley Foley, T. Kevin Hitchens, Song Li and Ke-Jie Yin**

Supporting Information

Genetic deficiency of microRNA-15a/16-1 confers resistance to neuropathological damage and cognitive dysfunction in experimental vascular cognitive impairment and dementia

Chao Zhou, Ping Sun, Yang Xu, Yuang Chen, Yixian Huang, Milton H. Hamblin, Lesley Foley, T. Kevin Hitchens, Song Li, Ke-Jie Yin[*]

C. Zhou, P. Sun, Y. Xu, K.J. Yin
Pittsburgh Institute of Brain Disorders & Recovery, Department of Neurology
University of Pittsburgh School of Medicine
Pittsburgh, PA, 15213, USA

K.J. Yin
Geriatric Research, Education and Clinical Center,
Veterans Affairs Pittsburgh Healthcare System,
Pittsburgh, PA, 15240, USA

Y. Chen, Y. Huang, S. Li
Center for Pharmacogenetics
University of Pittsburgh School of Pharmacy,
Pittsburgh, PA, 15213, USA.

M.H. Hamblin
Tulane University Health Sciences Center, Tulane University,
New Orleans, LA, 70112, USA

L. Foley, T. K. Hitchens.
Animal Imaging Center, Department of Neurobiology,
University of Pittsburgh School of Medicine,
Pittsburgh, PA, 15203, USA

***Correspondence addressed to:**

Ke-Jie Yin, M.D., Ph.D.
Pittsburgh Institute of Brain Disorders & Recovery, Department of Neurology,
University of Pittsburgh School of Medicine,
Pittsburgh, PA 15213
Email: yink2@upmc.edu

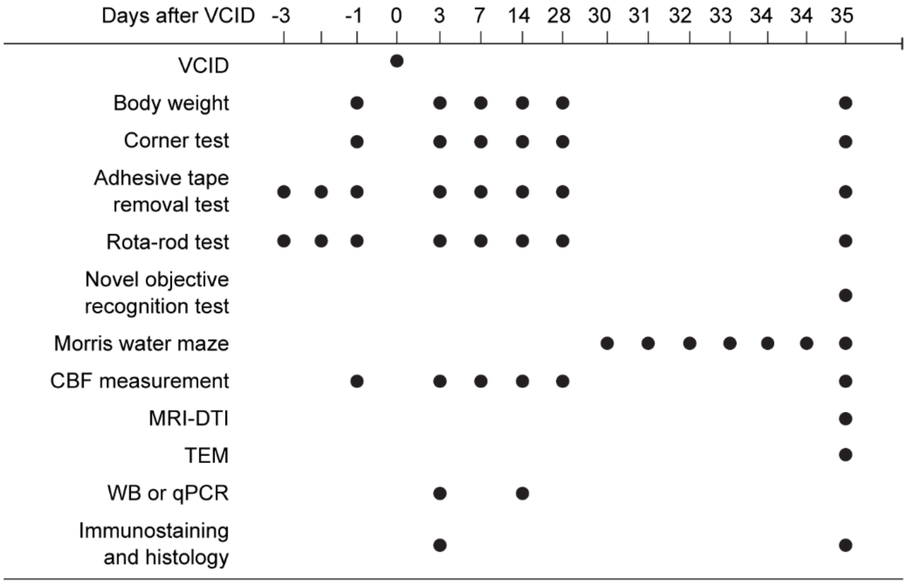

Figure S1. Schematic diagram of in vivo experimental design. Mice were subjected to sham or VCID operation. Cerebral blood flow (CBF) was measured with laser speckle imaging at 15 min before VCID, 3 d, 7 d, 14 d, 28 d, and 35 d after VCID. To detect sensorimotor and cognitive functions, neurobehavioral tests were performed in experimental mice up to 35 d after VCID. MRI and TEM were performed in mice 35 d after VCID to assess white matter and grey matter injury. Immunofluorescence and histological staining were conducted in brain sections in mice 3 d and 35 d after VCID to examine white and grey matter injury as well. Brain samples were harvested in experimental mice 3 d and 35 d after VCID for biochemical analysis. Related to Figure 1

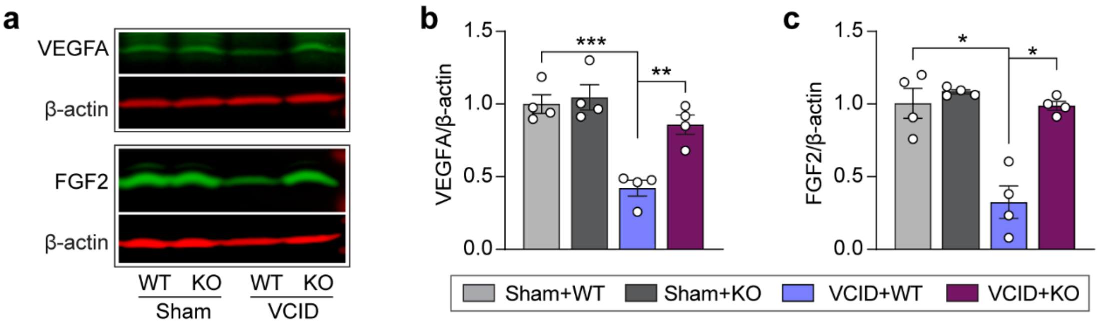

Figure S2. Genetic deletion of miR-15a/16-1 increases proangiogenic gene expression in mouse brains after 14 d of VCID. (**a**) Representative western blotting images of VEGFA, FGF2, and β-actin in the cerebral cortex. (**b**) Quantitative analysis showing VEGFA relative protein expression in the cerebral cortex (one-way ANOVA and Bonferroni's tests). (**c**) Quantitative analysis showing FGF2 relative protein expression in the cerebral cortex (Welch ANOVA test and Dunnett T3 test). Data are represented as mean ± SEM, n=4/group. *$p<0.05$, **$p<0.01$, or ***$p<0.001$ versus VCID+WT group.

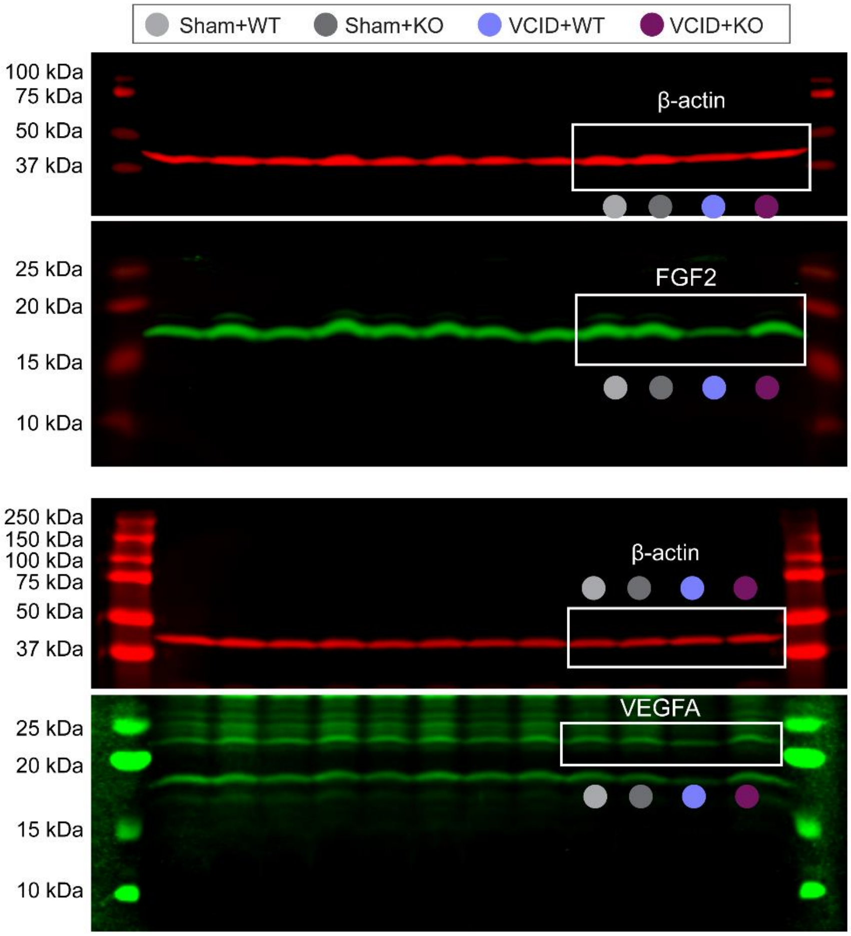

Figure S3. Western blotting raw data. Related to Figure S2.

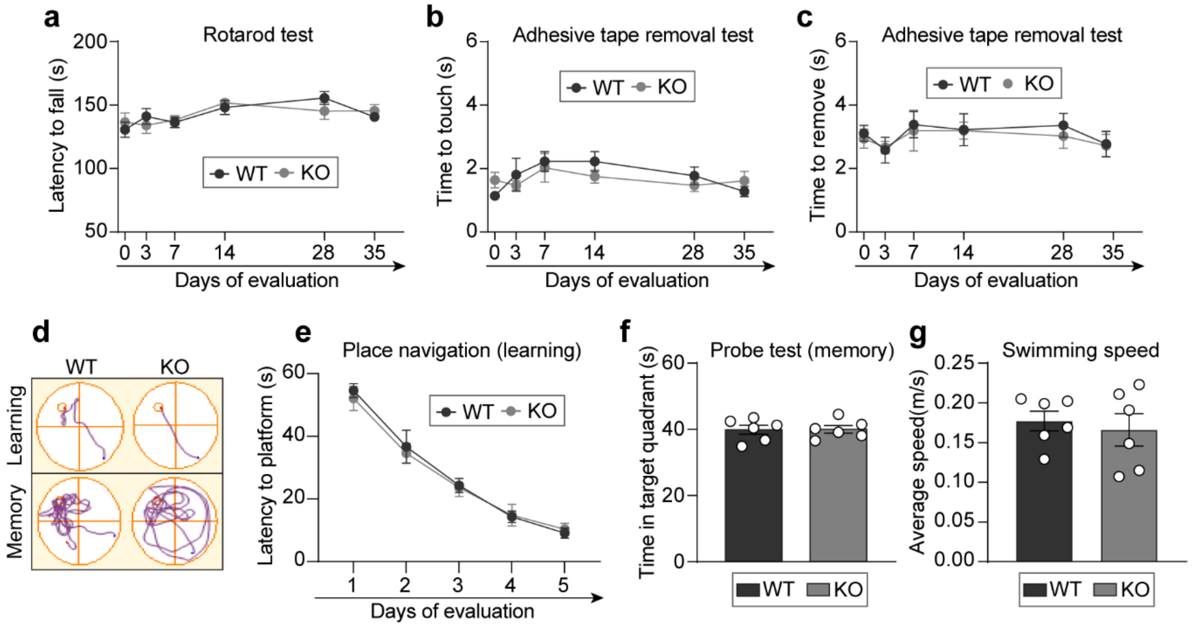

Figure S4. Genetic deletion of miR-15a/16-1 has no effect on neurobehavioral function in mice under physiological conditions. Long-term sensorimotor functions were evaluated in miR-15a/16-1 KO and WT mice by rotarod test and adhesive tape removal test. (**a**) The time to fall in the rotarod test. (**b**) The time to touch and (**c**) time to remove the tape in the adhesive tape removal test. Long-term cognitive function was evaluated in miR-15a/16-1 KO and WT mice by Morris water maze (MWM) test. **(d)** Swim paths of learning and memory phases during the MWM test. (**e**) The latency to find the hidden platform in the place navigation phase (learning). (**f**) The swim time in the target quadrant in the probe test (memory). (**g**) Average swimming speed in the MWM test. Data are presented as mean ± SEM, n=6/group. No significant difference in either sensorimotor or cognitive function was detected between WT and miR-15a/16-1 KO mice under normal conditions. Statistical analyses were performed by two-way ANOVA with Bonferroni's test (**a, b, c, e**) and unpaired t test **(f, g)**.

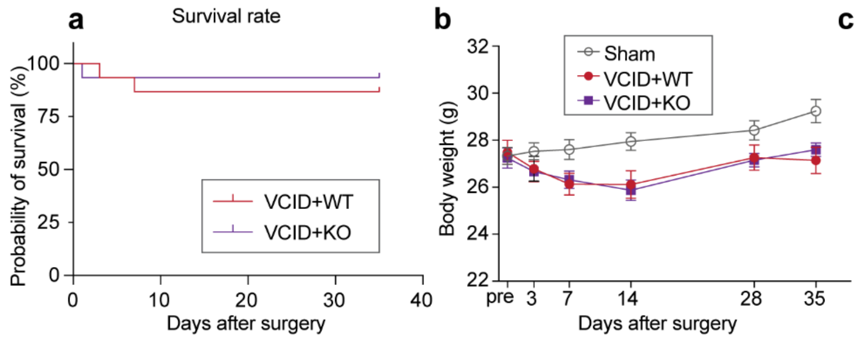

c

Days elapsed	VCID+WT	VCID+KO
0	100	100
1		93.33
3	93.33	
7	86.67	
35	86.67	93.33

d

Studies	Excluded animal #	Groups	Time points	Reasons
Neurobehavioral tsets	1	VCID+WT	Day 3	Death
	2	VCID+WT	Day 7	Death
	3	VCID+WT	Day 30	Blind due to CBF measurement, enthanized
	1	VCID+KO	Day 1	Death
Cerebral blood flow measurement	1	VCID+WT	Day 3	Death
	2	VCID+WT	Day 7	Death
	3	VCID+WT	Day 30	Blind due to CBF measurement, enthanized
	1	VCID+KO	Day 1	Death
Staining, WB and qPCR	1	VCID+WT	Day 3	Death
	2	VCID+WT	Day 7	Death
	3	VCID+WT	Day 30	Blind due to CBF measurement, enthanized
	1	VCID+KO	Day 1	Death

Figure S5. Sequential tracing of survival rate and body weight in miR-15a/16-1 knockout and WT mice after VCID. (**a**)The survival curve of miR-15a/16-1 KO and WT mice after VCID was traced, and each death event was recorded immediately. No difference in the survival rate was observed between the VCID+WT and VCID+KO groups, n=13-15 /group. (**b**) Body weights of miR-15a/16-1 KO and WT mice were measured before or 3-35 d after VCID. Body weights of sham-operated animals were also measured at indicated time points. MiR-15a/16-1 KO and WT mice showed similar patterns of body weight change after VCID. No significant difference was observed in miR-15a/16-1 KO and WT mice under sham-operated conditions. Data are shown as mean ± SEM, n = 10-15/group. Statistical analyses were performed by Log-rank (Mantel-Cox) test (p = 0.3196) and Gehan-Breslow-Wilcoxon test (p = 0.3255). (**c, d**) Tables showing all animals excluded from analyses.

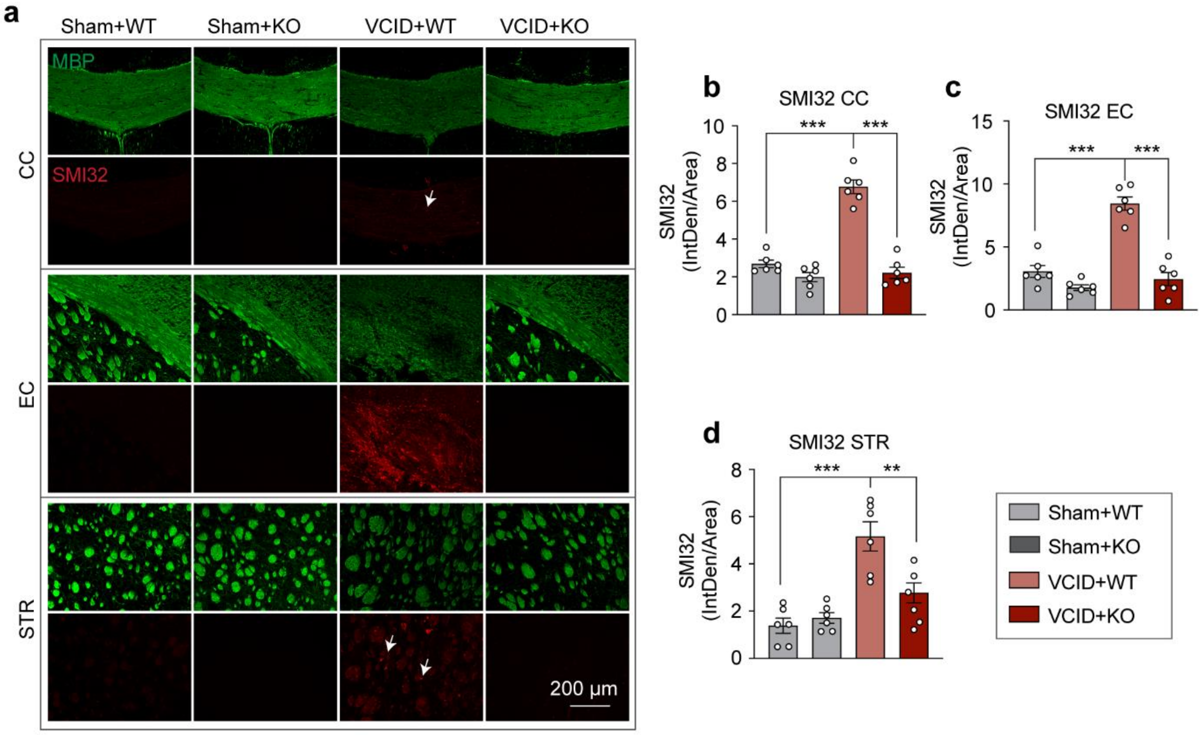

Figure S6. Genetic deletion of miR-15a/16-1 reduces axonal damage after 35 d of VCID. (**a**) Representative immunofluorescence staining images of MBP (green) and SMI32 (red) in the corpus callosum (CC), external capsule (EC), and striatum (STR) areas (white arrow: damaged axons). (**b-d**). Quantitative analysis of SMI32 mean fluorescence intensities in the CC, EC, and STR areas showing less axonal damage in miR-15a/16-1 KO mice 35 d after VCID compared with WT controls. Data are represented as mean ± SEM, n=6/group. *p<0.05, **p<0.01, or ***p<0.001 versus VCID+WT group. Statistical analyses were performed by one-way ANOVA and Bonferroni's tests. Related to Figure 3

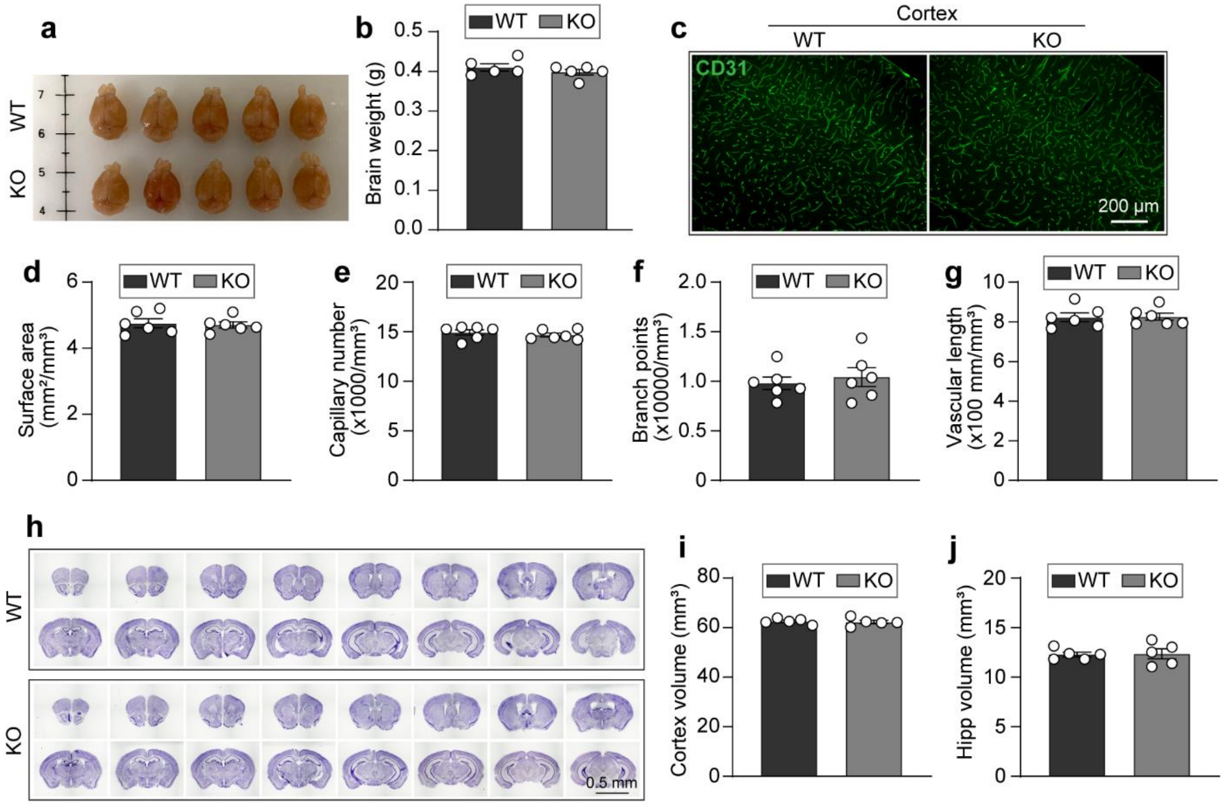

Figure S7. Genetic deletion of miR-15a/16-1 has no effects on mouse brain anatomy and morphology under normal conditions. (a) Brain gross specimens of miR-15a/16-1 KO and WT mice (n=5/group). **(b)** Quantitative analysis of brain weight (n=5/group). **(c)** Representative CD31 immunostaining in the cerebral cortex area. Quantitative analysis of **(d)** surface area, **(e)** capillary number, **(f)** branch points, and **(g)** vascular length of miR-15a/16-1 KO and WT mice under normal conditions (n=6/group). **(h)** Representative images of Cresyl Violet staining. (**i, j**) Quantitative analysis of volume in the cerebral cortex (CTX) and hippocampus (Hipp) areas from miR-15a/16-1 KO and WT mice under normal conditions (n=5/group). Data are presented as mean ± SEM. Statistical analyses were performed by unpaired t test.

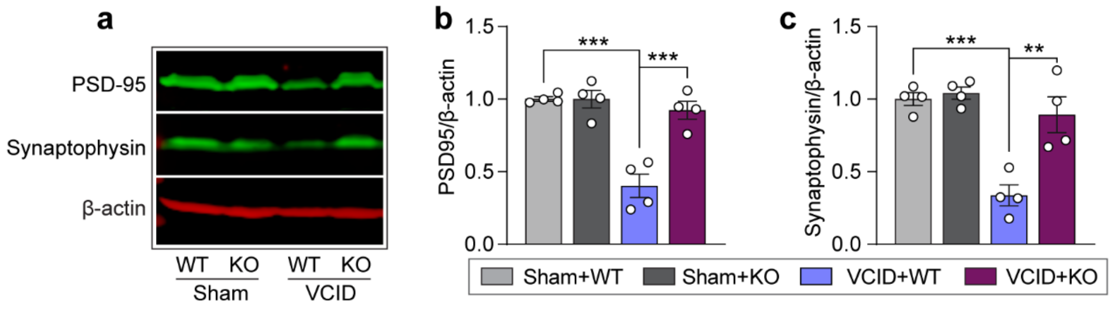

Figure S8. Genetic deletion of miR-15a/16-1 increases synaptic plasticity-related proteins PSD-95 and synaptophysin in mouse brains after 14 d of VCID. (**a**) Representative western blotting images of PSD-95, synaptophysin, and β-actin in mouse cerebral cortex. (**b**) Quantitative analysis showing PSD-95 relative protein expression in the cerebral cortex (one-way ANOVA and Bonferroni's tests). (**c**) Quantitative analysis showing synaptophysin relative protein expression in the cerebral cortex (one-way ANOVA and Bonferroni's tests). Data are represented as mean ± SEM, n=4/group. *p<0.05, **p<0.01, or ***p<0.001 versus VCID+WT group.

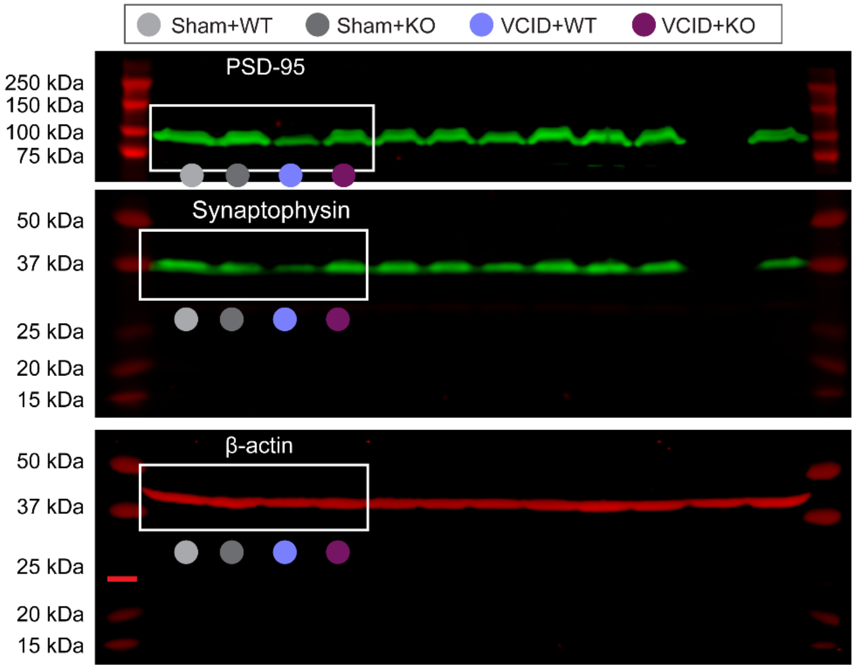

Figure S9. Western blotting raw data. Related to Figure S8.

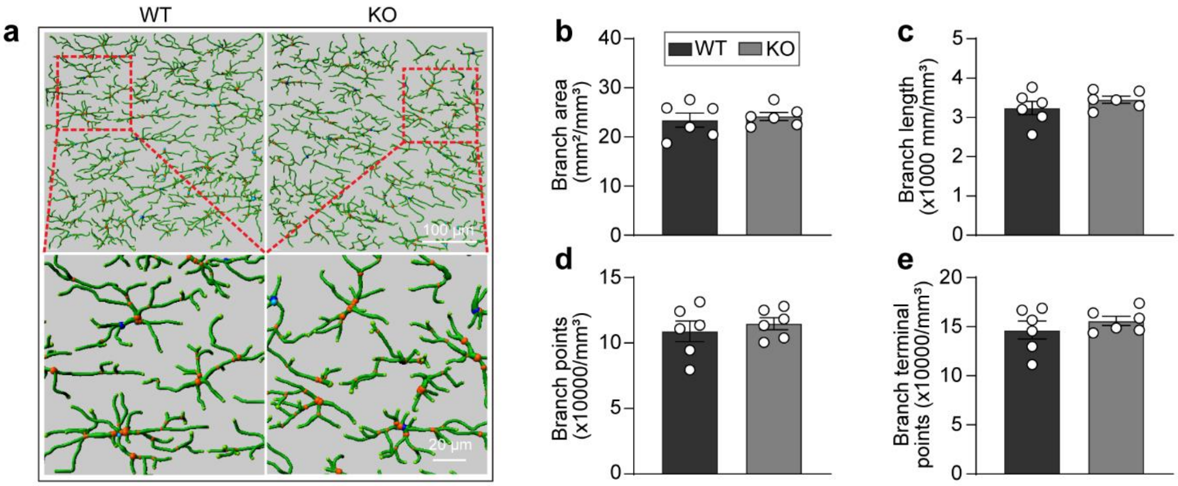

Figure S10. Genetic deletion of miR-15a/16-1 has no effect on astrocytic morphology in mouse brains under physiological conditions. (a) Representative images of astrocytic skeleton that were generated by Imaris software based on GFAP immunostaining. **(b)** Quantitative analysis of astrocytic branch area, **(c)** branch length, **(d)** branch points, and **(e)** branch terminal points. Under normal conditions, no significant difference for astrocytic morphology was detected in brains of miR-15a/16-1 KO mice compared with WT controls. Data are presented as mean ± SEM, n=6/group. Statistical analyses were performed by unpaired t test.

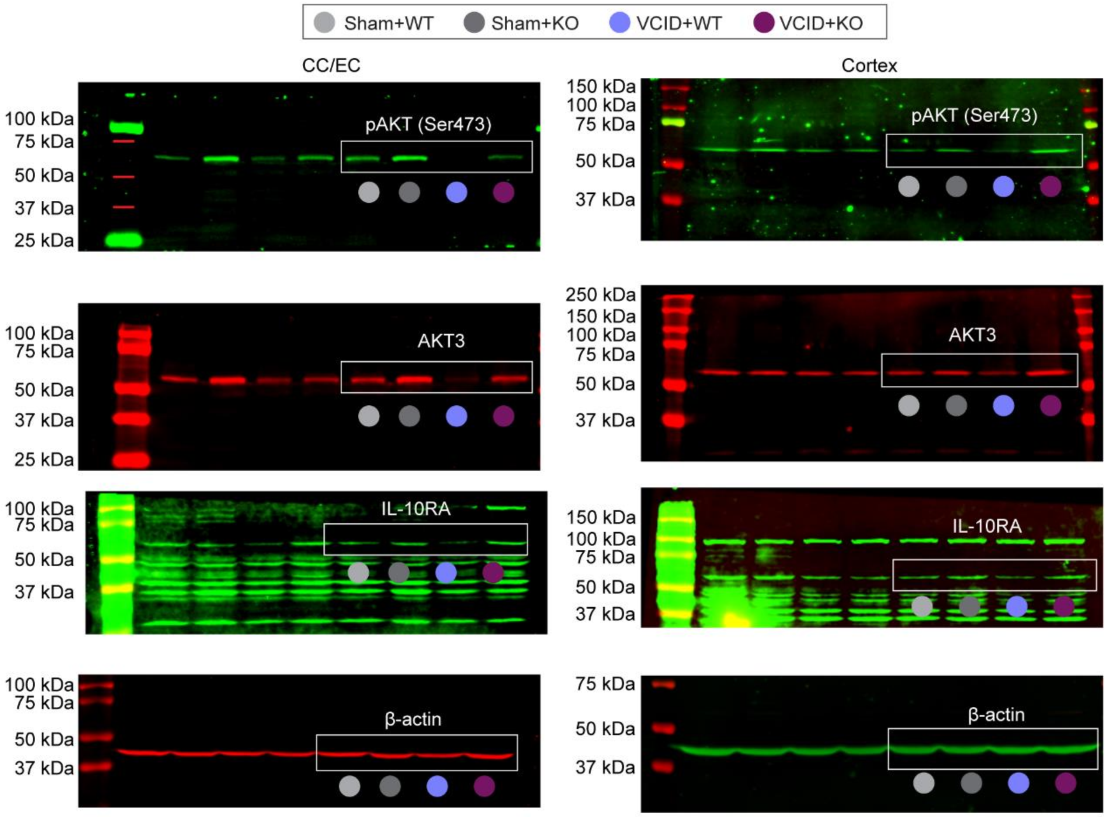

Figure S11. Western blotting raw data. Related to Figure 6.

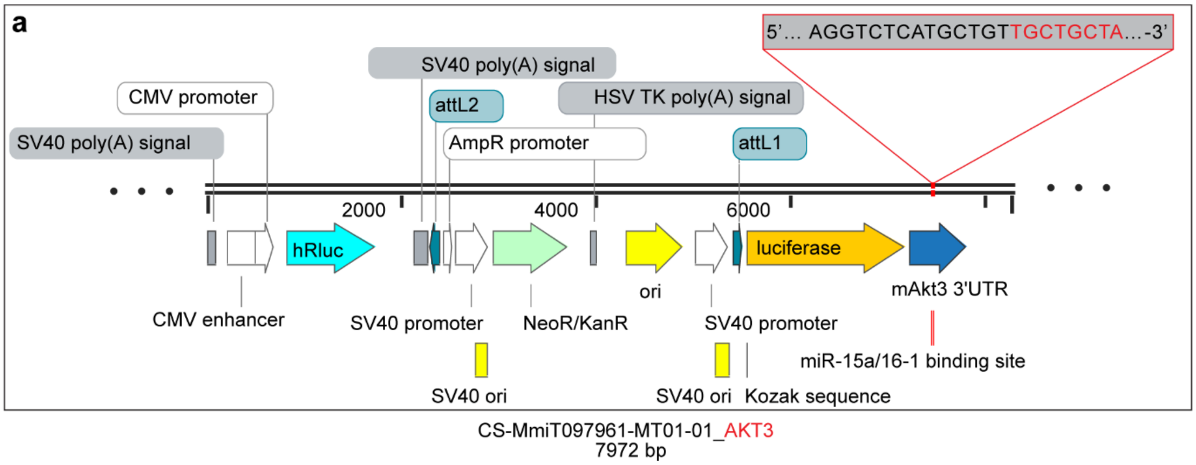

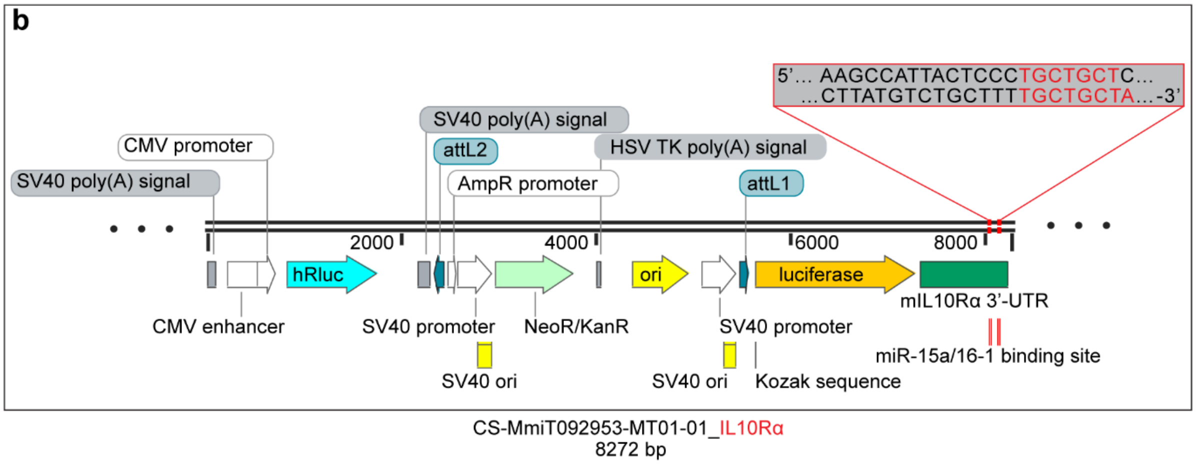

Figure S12. Structures of plasmids used in dual-luciferase reporter assays. A Firefly/Renilla dual-luciferase reporter vector (pEZX-MT01, Genecopoeia) was utilized to construct 3'-UTR fragments of miR-15a/16-1 target genes. (**a**) Mouse AKT3 3'-UTR dual-luciferase reporter plasmid (pEZX-MT01-mAKT3 3'-UTR) contains a 600-bp fragment of the 3'-UTR sequence of mouse AKT3 mRNA and one miR-15a/16-1 binding site (shown in red color, 8 bp). The mutated plasmid (pEZX-MT02-mAKT3 3'-UTR) carries a 600-bp mouse AKT3 3'-UTR sequence in which the miR-15a/16-1 binding site was mutated. Similarly, (**b**) the mouse IL-10RA 3'-UTR dual-luciferase reporter plasmid (pEZX-MT01-mIL-10RA 3'-UTR) contains a 900-bp fragment of the 3'-UTR sequence of mouse IL-10RA mRNA and carries two putative miR-15a/16-1 binding sites (shown in red color, 7 bp and 8 bp). The mutated plasmid (pEZX-MT02-mIL-10RA 3'-UTR) contains a 900-bp fragment of the 3'-UTR sequence of mouse IL-10RA mRNA in which two miR-15a/16-1 binding sites were mutated. Related to Figure 6

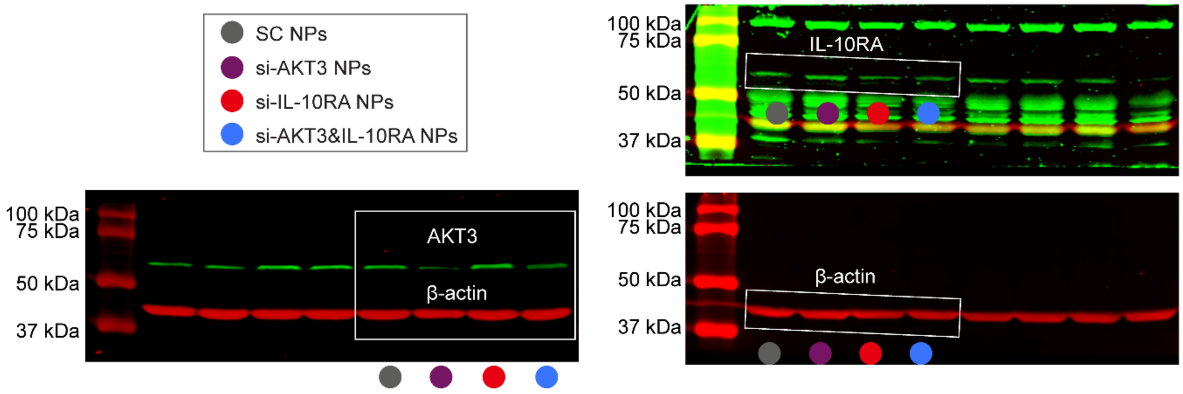

Figure S13. Western blotting raw data. Related to Figure 9

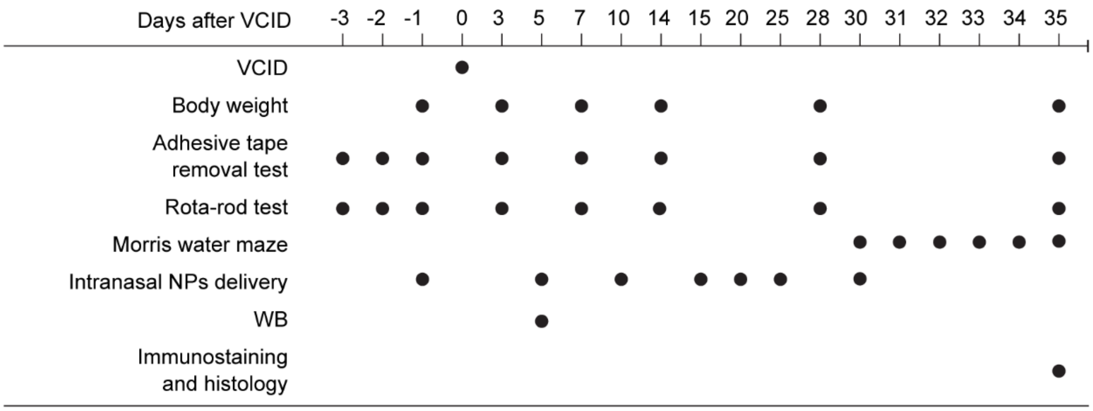

Figure S14. Experimental design for intranasal delivery of siRNA-loaded nanoparticles. MiR-15a/16-1 KO and WT mice were subjected to a VCID operation and intranasal delivery of AKT3 and IL-10RA siRNA-loaded nanoparticles before and 3 d, 7 d, 14 d, 28 d, and 35 d after VCID. Gene silencing efficiency of siRNA-loaded-NPs was evaluated at 5 d following administration. Sensorimotor function was evaluated in experimental mice before and 3 d, 7 d, 14 d, 28 d, and 35 d after VCID. Cognitive function was evaluated in mice 30-35 d after VCID. After neurobehavioral testing, mice were sacrificed and brain sections were harvested to assess white matter and grey matter injury. Related to Figure 8

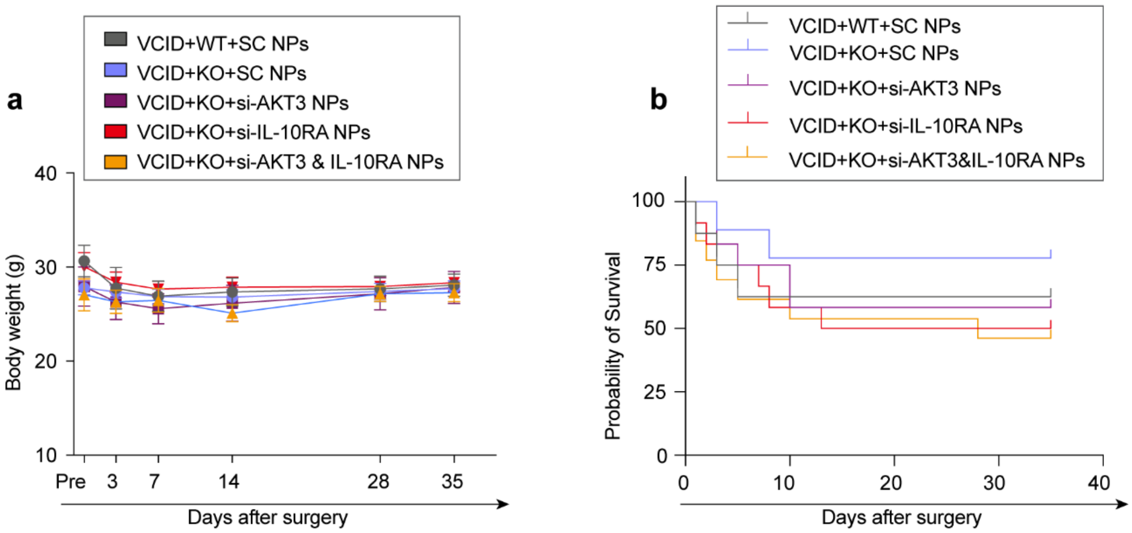

c

Days elapsed	WT+SC NPs	KO+SC NPs	KO+si-AKT3 NPs	KO+si-IL-10RA NPs	KO+si-AKT3&IL-10RA NPs
0	100	100	100	100	100
1	87.50			91.67	84.62
2				83.33	76.92
3	75.00	88.89	83.33	75.00	69.23
5	62.50		75.00		61.53
7				66.67	
8		77.78		58.33	
10			58.33		53.85
13				50.00	
28					46.15
35	62.50	77.78	58.33	50.00	46.15

Figure S15. Sequential tracing of the survival rates and body weights following intranasal delivery of AKT3 and/or IL-10RA siRNA-loaded nanoparticles in miR-15a/16-1 KO mice and WT controls. (**a**) Body weights of miR-15a/16-1 KO and WT mice were measured before or 3-35 d after VCID. Experimental mice in all groups showed similar patterns of body weight changes after VCID and NP administration. No significant differences were observed among the different groups (n=9-15/group, two-way ANOVA with Bonferroni's test). (**b**) The survival curve of miR-15a/16-1 KO and WT mice after VCID and administration of NPs were traced, and each death event (**c**) was recorded immediately. No significant differences in survival rates were observed among the different groups. Data are shown as mean ± SEM, n=9-15/group. Statistical analyses were performed by Log-rank (Mantel-Cox) test and Gehan-Breslow-Wilcoxon test among different groups. Related to Figure 8

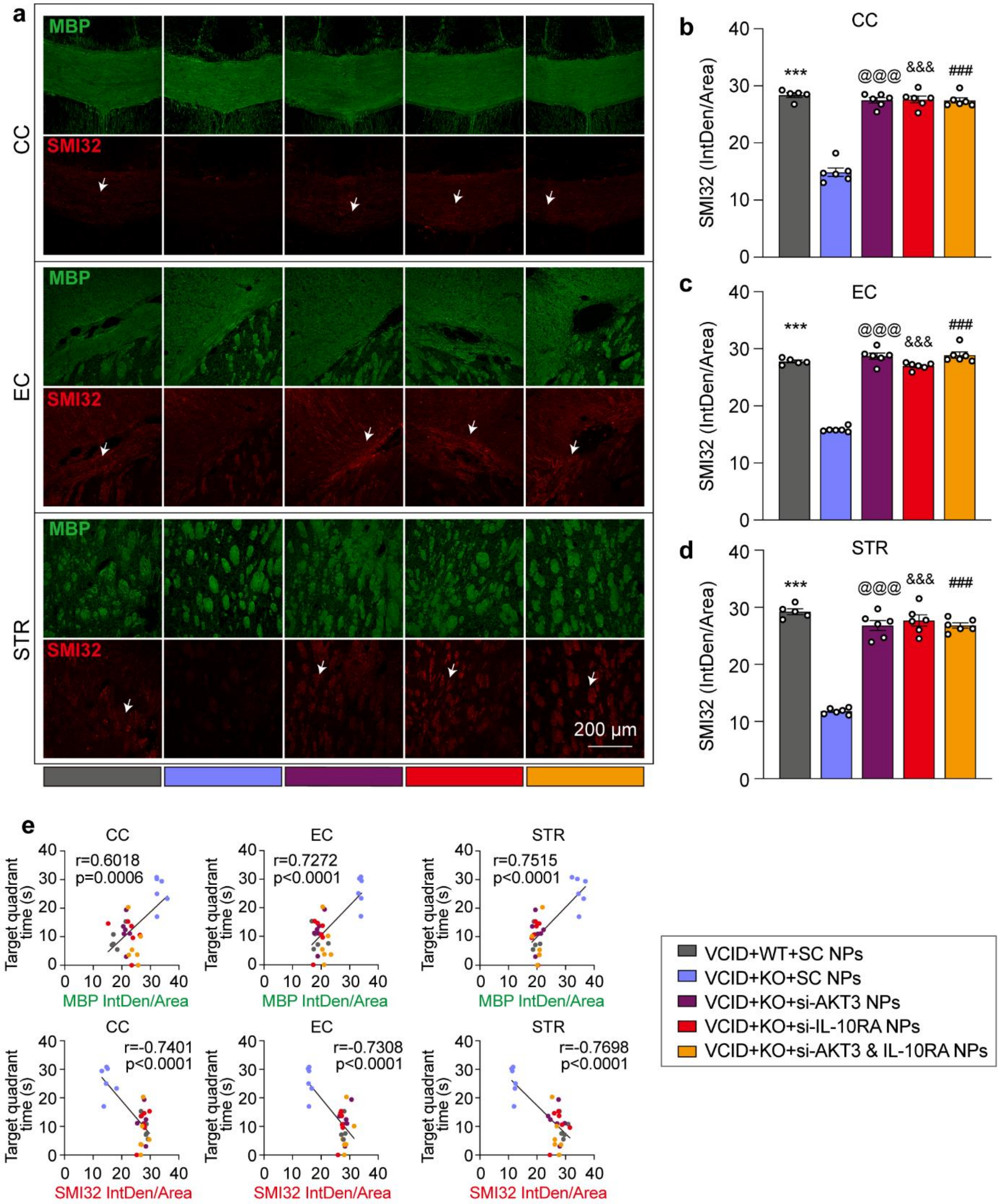

Figure S16. Knockdown of cerebral AKT3 and IL-10RA increases axonal damage in miR-15a/16-1 KO mice after VCID. (**a**) Representative immunofluorescence staining images of MBP (green) and SMI32 (red) in the CC, EC, and STR areas (white arrow: damaged axons). (**b-**

d). Quantitative analysis of SMI32 mean fluorescence intensity in the CC, EC, and STR areas indicates increased post-VCID axonal damage in miR-15a/16-1 KO mice after intranasal delivery of AKT3 and/or IL-10RA-siRNA-loaded NPs. Data are represented as mean ± SEM, n=5-6/group. *, $^{@}$, $^{\&}$, $^{\#}$ $p < 0.05$; **, $^{@@}$, $^{\&\&}$, $^{\#\#}$ $p < 0.01$; and ***, $^{@@@}$, $^{\&\&\&}$, $^{\#\#\#}$ $p < 0.001$ versus VCID+KO+SC NPs group. Statistical analyses were performed by one-way ANOVA and Bonferroni's test. (**e**) Correlation analyses of MBP or SMI32 mean fluorescence intensity with cognitive function (target quadrant time in the Morris water maze test), showing that white matter damage correlated with impaired cognitive function in miR-15a/16-1 KO mice administrated AKT3 and/or IL-10RA siRNA-loaded NPs (n=5-6/group, two-tailed Pearson correlation analysis). Related to Figure 9

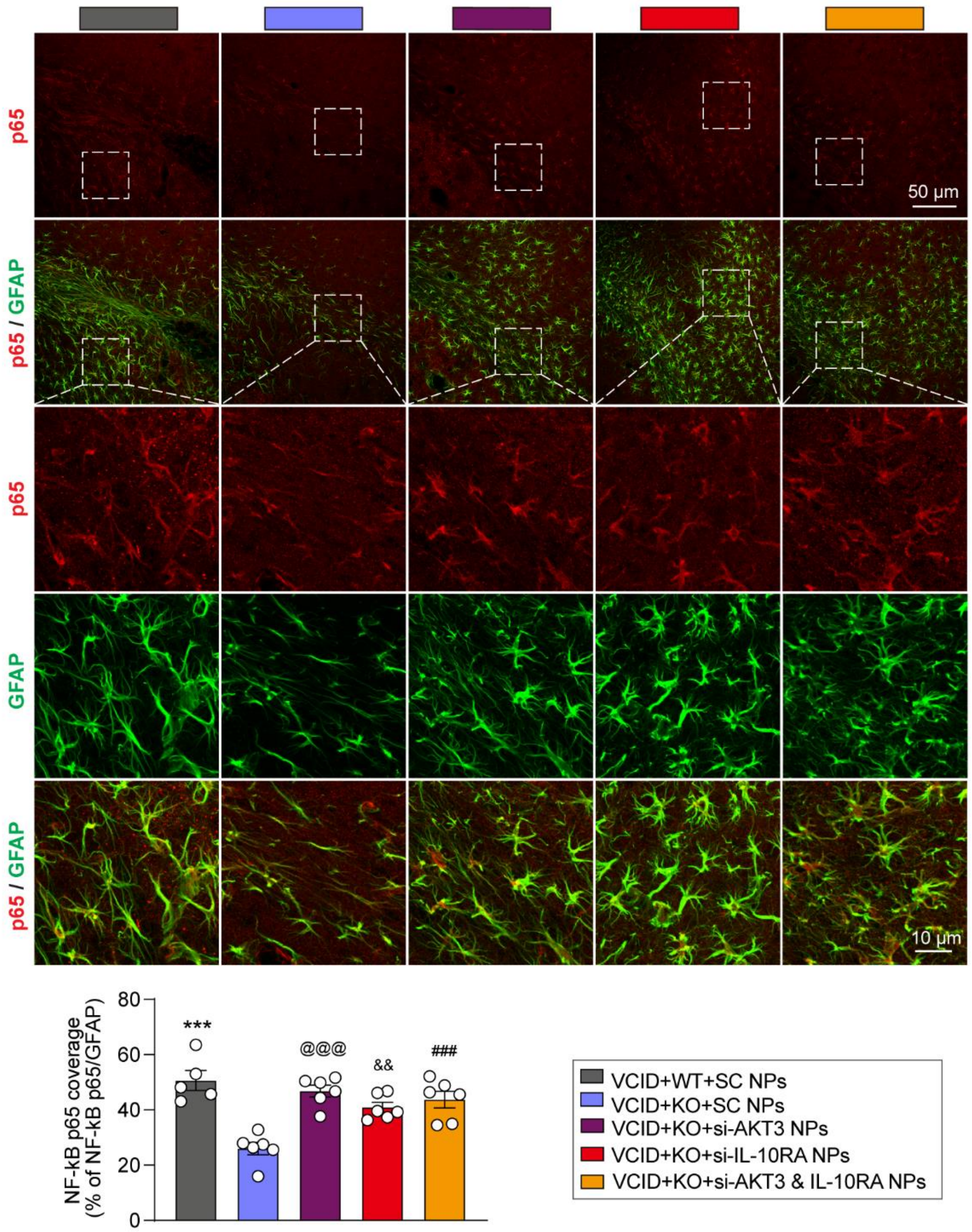

Figure S17. Knockdown of cerebral AKT3 and IL-10RA increases astrocytic activation of NF-kB p65 in miR-15a/16-1 KO mice after VCID. (**a**) Representative immunofluorescence staining images of GFAP (green) and NF-kB p65 (red). (**b**) Quantitative analysis of NF-kB p65 coverage indicates increased post-VCID NF-kB p65 activation in astrocytes after intranasal delivery of AKT3 and/or IL-10RA-siRNA-loaded NPs in miR-15a/16-1 KO mice. Data are represented as mean ± SEM, n=5-6/group. *, $^{@}$, $^{\&}$, $^{\#}$ $p < 0.05$; **, $^{@@}$, $^{\&\&}$, $^{\#\#}$ $p < 0.01$; and ***, $^{@@@}$,

$^{\&\&\&}$, $^{\#\#\#}$ $p < 0.001$ versus VCID+KO+SC NP group. Statistical analyses were performed by one-way ANOVA and Bonferroni's test.

Table S1. List of primary antibodies used in this study

Antibodies	Host species	WB dilution	IF staining dilution	Companies	Catalog #
AKT3 (E1Z3W)	Rabbit	1:1000	1:200	Cell Signaling	14982s
p-AKT (Ser473)	Rabbit	1:500		Cell Signaling	4060
IL-10RA	Rabbit	1:500		Invitrogen	PA5-109852
β-actin	Mouse	1:2000		Sigma-Aldrich	A5441
VEGFA	Rabbit	1:500		Abcam	Ab51745
FGF2	Rabbit	1:800		Sigma-Aldrich	SAB2108135
PSD95	Rabbit	1:500		Cell Signaling	2507
Synaptophysin	Rabbit	1:500		Cell Signaling	36406
NF-κB p65	Rabbit		1:200	Abcam	Ab16502
APC	Mouse		1:200	EMD Millipore	OP80
AKT3	Mouse		1:200	Invitrogen	MA141201
CD31	Rat		1:200	BD Biosciences	553370
MBP	Rabbit		1:500	Abcam	Ab40390
SMI32	Mouse		1:500	Biolegend	801701
GFAP	Goat		1:500	Abcam	Ab53554
GFAP	Rabbit		1:500	Sigma-Aldrich	G9269
GFP-GFAP	Mouse		1:1000	EMD Millipore	MAB3402X
Iba-1	Goat		1:500	Abcam	Ab5076
Iba-1	Rabbit		1:500	Wako	019-19741
NeuN	Mouse		1:500	EMD Millipore	MAB377
NeuN	Rabbit		1:500	EMD Millipore	ABN78

Table S2. List of primers used in this study

Genes	Primer sequences	Annealing temperatures	Amplicon sizes
AKT3	Forward: 5’- TGGGTTCAGAAGAGGGGAGAA -3’<br>Reverse: 5’-AGGGGATAAGGTAAGTCCACATC-3’	59°C	122 bp
IL-10RA	Forward: 5’-CCCATTCCTCGTCACGATCTC-3’<br>Reverse: 5’-TCAGACTGGTTTGGGATAGGTTT-3’	59°C	141 bp
Cyclophilin	Forward: 5’-ACTCCTCATTTAGATGGGCATCA-3’<br>Reverse: 5’-GAGTATCCGTACCTCCGCAAA-3’	59°C	126 bp
